# Antipsychotic dose, dopamine D2 receptor occupancy and extrapyramidal side-effects: a systematic review and dose-response meta-analysis

**DOI:** 10.1038/s41380-023-02203-y

**Published:** 2023-08-03

**Authors:** Spyridon Siafis, Hui Wu, Dongfang Wang, Angelika Burschinski, Nobuyuki Nomura, Hiroyoshi Takeuchi, Johannes Schneider-Thoma, John M. Davis, Stefan Leucht

**Affiliations:** 1 Department of Psychiatry and Psychotherapy, School of Medicine, Technical University of Munich, Munich, Germany; 2 Department of Neuropsychiatry, Keio University School of Medicine, Tokyo, Japan; 3 Psychiatric Institute, University of Illinois at Chicago, Chicago, IL, USA; 4 Maryland Psychiatric Research Center, Baltimore, MD, USA

**Keywords:** Schizophrenia, Neuroscience

## Abstract

Antipsychotic drugs differ in their propensity to cause extrapyramidal side-effects (EPS), but their dose-effects are unclear. Therefore, we conducted a systematic review and dose-response meta-analysis. We searched multiple electronic databases up to 20.02.2023 for fixed-dose studies investigating 16 second-generation antipsychotics and haloperidol (all formulations and administration routes) in adults with acute exacerbations of schizophrenia. The primary outcome was the number of participants receiving antiparkinsonian medication, and if not available, the number of participants with extrapyramidal side-effects (EPS) and the mean scores of EPS rating scales were used as proxies. The effect-size was odds ratio (ORs) compared with placebo. One-stage random-effects dose-response meta-analyses with restricted cubic splines were conducted to estimate the dose-response curves. We also examined the relationship between dopamine D_2_ receptor (D_2_R) occupancy and ORs by estimating occupancies from administrated doses. We included data from 110 studies with 382 dose arms (37193 participants). Most studies were short-term with median duration of 6 weeks (range 3–26 weeks). Almost all antipsychotics were associated with dose-dependent EPS with varied degrees and the maximum ORs ranged from OR = 1.57 95%CI [0.97, 2.56] for aripiprazole to OR = 7.56 95%CI [3.16, 18.08] for haloperidol at 30 mg/d. Exceptions were quetiapine and sertindole with negligible risks across all doses. There was very low quality of findings for cariprazine, iloperidone, and zotepine, and no data for clozapine. The D_2_R occupancy curves showed that the risk increased substantially when D_2_R occupancy exceeded 75–85%, except for D_2_R partial agonists that had smaller ORs albeit high D_2_R occupancies. In conclusion, we found that the risk of EPS increases with rising doses and differs substantially in magnitude among antipsychotics, yet exceptions were quetiapine and sertindole with negligible risks. Our data provided additional insights into the current D_2_R therapeutic window for EPS.

## Introduction

Extrapyramidal side-effects (EPS) or drug-induced movement disorders, such as parkinsonism, dystonia, akathisia, and tardive dyskinesia [1], are among the most common side-effects of antipsychotic medications that can be present in up to a third of people with schizophrenia [2]. They can be stigmatizing and unpleasant, leading to nonadherence to treatment and the subsequent negative impacts [3]. Moreover, they often require the use of adjunctive medications, such as anticholinergic medications, which could further increase the side-effect burden, e.g., cognitive impairment and constipation [4]. Therefore, proper information about the risk of EPS associated with antipsychotic treatment is necessary.

The principal pathogenetic mechanism of antipsychotic-induced EPS is the blockade of the dopamine D_2_ receptor (D_2_R) signalling in the nigrostriatal pathway [5]. All current antipsychotics bind to the D_2_R, albeit with different affinities and receptor-binding profiles [6–8]. According to the receptor occupancy theory, the D_2_R occupancy of an antipsychotic depends on its affinity to the D_2_R and the plasma concentration [9], which is associated with the administrated dose [10]. Antipsychotic drugs typically reach maximum efficacy at doses corresponding to approximately 80% D_2_R occupancy, with higher doses beyond this threshold increasing the risk of EPS [8, 11–17]. However, the exact mechanism is more complex, and other receptors, including serotonin 5-HT_1A_, 5-HT_2A_, 5-HT_2c_ and muscarinic M_1_ receptors, also play a role [8, 18–20]. Therefore, the risk of EPS varies among antipsychotics, with newer or “second-generation” antipsychotics generally having a lower risk [21].

While EPS may be dose-dependent, the dose-response curves remain unclear and can differ across medications [22]. Additionally, while the above-mentioned D_2_R therapeutic window has been documented in molecular imaging studies [8, 12–14], it has not yet been comprehensively evaluated in systematic reviews of clinical trials. To further elucidate these issues, we conducted a comprehensive systematic review and dose-response meta-analysis on antipsychotic-induced EPS.

## Methods and materials

We followed the PRISMA statement (eAppendix 1) [23], pre-registered the protocol (PROSPERO-ID: CRD42020181467, data extraction for this analysis had started before submission of the protocol), and noted any deviations (eAppendix 2).

### Eligibility criteria

#### Participants

We included adults with acute exacerbations of schizophrenia spectrum disorders, i.e., schizophrenia, schizoaffective and schizophreniform disorder, without other restrictions in terms of age, sex, ethnicity, setting, previous response to treatment and diagnostic criteria. We analysed separately studies focusing on predominant negative symptoms, first-episode, and elderly, given that these patients may require lower doses and could be more vulnerable to side-effects [24]. We excluded studies on stable patients (relapse prevention studies) due to methodological and clinical heterogeneity, e.g., pre-exposure to antipsychotics in the stabilization phase.

#### Interventions

We included studies evaluating monotherapy with 16 second-generation antipsychotics, i.e., amisulpride, aripiprazole, asenapine, brexpiprazole, cariprazine, clozapine, iloperidone, lumateperone, lurasidone, olanzapine, quetiapine, paliperidone, risperidone, sertindole, ziprasidone, and zotepine, the first-generation antipsychotic haloperidol (commonly used as an active comparator), and placebo. Studies allocating participants to fixed-dosing schedules or narrow fixed dose ranges were eligible, and flexible-dosing schedules were excluded. There was no restriction in terms of formulations (e.g., oral, long-acting intramuscular injection, transdermal, and immediate- and extending-release). Different formulations were combined in the primary analysis by converting doses to daily oral equivalents similar to our previous analysis [25]. Nevertheless, they were also analysed separately in a sensitivity analysis.

#### Study design

We included open and blinded randomized-controlled trials (RCTs) with a minimum study duration of 3 weeks [26] comparing fixed-doses of the above-mentioned antipsychotics or placebo in people with acute exacerbation of schizophrenia. Nevertheless, we excluded studies that investigated only head-to-head comparisons between two different antipsychotics, as well as relapse-prevention studies. We also excluded studies with a high risk of bias in terms of randomization [27]. In case of crossover trials, we used the first phase in order to avoid carry-over effects [28]. Cluster-randomized trials were excluded because of unit-of-analysis problems [29].

#### Search strategy

We searched up to 06.03.2022 the study-based trial register of the Cochrane Schizophrenia Group [30], which included regular searches in multiple electronic databases and hand searches. We also conducted update searches up to 20.02.2023 in PubMed and CENTRAL. Moreover, we inspected reference lists of previous reviews on relevant topics (search strings and details provided in eAppendix 3).

#### Outcomes

We considered a priori three outcomes: (1) mean change scores of validated scales measuring EPS, e.g., preferably with the Simpson and Angus Scale (SAS) [31], (2) number of participants that received at least once antiparkinsonian medication, and (3) number of participants with at least one EPS.

The continuous data on scale scores were heavily skewed, and thus, we did not use them as the primary outcome. Moreover, in order to allow a more comprehensive synthesis with increased power, we defined our primary outcome *post-hoc* as dichotomous using data from the number of patients receiving antiparkinsonian medication, and if not available, data from the number of patients with at least one EPS and mean change scores of rating scales (see also below “Data synthesis”). Nevertheless, the findings for the three aforementioned outcomes were also reported separately as sensitivity analyses.

### Data extraction and risk of bias assessment

Two of the reviewers (HW, SS, NN, DW, SL) independently selected studies, extracted data into a Microsoft Access database [32], and evaluated the risk of bias of individual studies using the Cochrane Risk of Bias tool 1 [27]. Discrepancies were solved by consensus, and study authors were contacted for further clarifications.

For continuous outcomes, we preferred change over endpoint data, and results accounting for missing outcome data over completer or per-protocol, giving preference to mixed-models of repeated measurement (MMRM) and multiple imputation over last-observation carried forward (LOCF). In case of missing standard deviations (SDs), they were calculated from standard errors, other test statistics, or imputed from the SDs of other studies [27, 33]. If only completer analyses were presented for dichotomous outcomes, we assumed that patients lost to follow-up did not have the outcome.

### Data synthesis

The effect sizes for continuous outcomes were standardized mean differences (SMDs) because different EPS scales were expected, and for dichotomous were odds ratios (ORs) because of their preferred mathematical properties [34, 35]. We also transformed SMDs to ORs using the Hasselblad and Hedges’ method [36–38], in order to allow the combination of continuous and dichotomous data for the primary outcome, as well as comparability of the findings in a sensitivity analysis. Placebo or 0 mg was used as reference in the calculation of effect-sizes. ORs were interpreted as small (OR = 1.52), medium (OR = 2.74) and large (OR = 4.72), assuming a risk of 5% in the placebo group (eAppendix 2) [39].

We estimated dose-response curves for each antipsychotic separately with a one-stage random-effects dose-response meta-analysis in a frequentist framework [40]. We used restricted cubic splines with three knots, which were set at the 25th, 50th and 75th percentiles of the doses, except for asenapine, at 10th, 50th and 90th percentiles, because the former percentiles could not form three knot points. We estimated the maximum risk for EPS and the corresponding dose. Dose-response curves were evaluated descriptively, as well as with a Wald test and the coefficients of the model [41]. Heterogeneity was quantified with the variance partition coefficient (VPC), which is a multivariate extension of the I^2^ statistic [40].

Patient subgroups were analysed separately and the robustness of the results were evaluated in predefined sensitivity analyses: (i) excluding non-dose-finding studies, i.e., single dose arm versus placebo, (ii) excluding studies with treatment-resistant patients, (iii) excluding open studies, (iv) analysing different formulations separately, (v) using knot points at 10th, 50th and 95th percentiles, and (vi) analysing separately the three outcome measures of EPS.

Furthermore, we *post-hoc* explored the relationship between D_2_R occupancy and the risk of EPS (eAppendix 2). We converted the dose-response curves of individual antipsychotics to occupancy-response curves by estimating the median D_2_R occupancy from the daily dose using Michaelis-Menten models derived from the previous meta-analysis of Lako et al 2013 (see eAppendix 2 for the formulas and their limitations) [11]. The meta-analysis of Lako et al 2013 provided formulas for eight antipsychotics, i.e., amisulpride, aripiprazole, clozapine, haloperidol, olanzapine, quetiapine, risperidone, and ziprasidone [11]. Moreover, we conducted a dose-response meta-analysis by combining the estimated D_2_R occupancies of the above-mentioned antipsychotics, except for aripiprazole that is a D_2_R partial agonist [42]. In this analysis, we set the knot points at the 25^th^, 50^th^ and 75^th^ percentiles of D_2_R occupancies >50%, given that we expected changes at this part of the curve [43].

We evaluated small-study effects and the potentially associated publication bias when there were at least 10 studies available with funnel plots of the pairwise comparison between an antipsychotic (any dose) versus placebo, and with dose-response meta-regression for sample size (eAppendix 2).

Quality of the evidence was evaluated with the GRADE approach [44], which was adapted to a dose-response meta-analysis and considering the domains of risk of bias, reporting bias, indirectness, inconsistency, and imprecision (eAppendix 9). The results of the assessments were summarized into four categories, i.e., high, moderate, low and very low confidence of evidence.

Data analysis was conducted with meta v5.1-1 [45] and dosresmeta v2.0.1 [41] in R statistical software v4.0.3 [46].

## Results

**Fig. 1:**
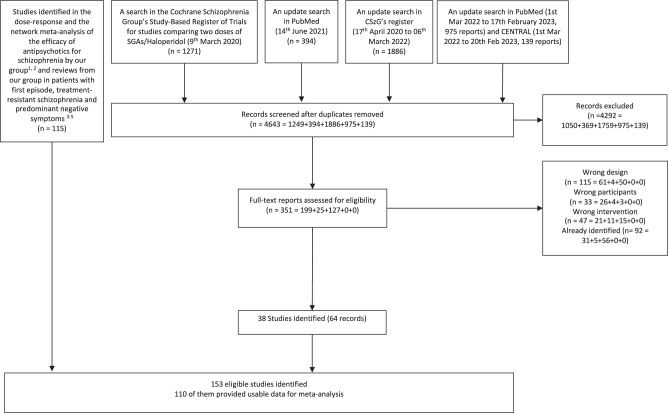
PRISMA flow diagram. The flow diagram shows the study selection process.

We included 110 studies with 382 dose arms and 37193 participants in this meta-analysis. The PRISMA diagram of the search is provided in Fig. 1. The table of study characteristics is presented in eAppendix 4. There were no eligible studies for the elderly subgroup, and sparse data on the subgroups of predominant negative symptoms (amisulpride and olanzapine) and first-episode (risperidone). We had data for all antipsychotics of interest, except for clozapine. The median study duration was 6 weeks (3–26 weeks). The participants had a mean age of 38.8 years old and around a third of them were female. For overall risk of bias of included studies, 66 studies were rated as low, 37 moderate, and 7 high. Summary of risk of bias assessments is provided (eAppendix 5).

### Dose-response curves of antipsychotic-induced extrapyramidal side-effects

**Fig. 2:**
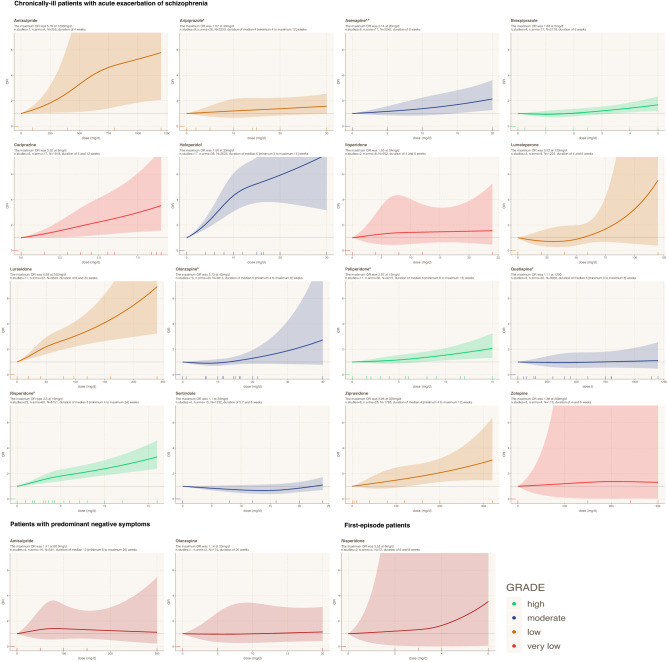
Dose-response curves of 16 antipsychotics for extrapyramidal side-effects. This figure shows the dose-response curves of 16 individual antipsychotics for extrapyramidal side-effects (EPS) in different subgroups of patients with schizophrenia. The X-axis displays the daily antipsychotic dose (mg/d), while the Y-axis displays the odds ratios (ORs) for the risk of EPS associated with a specific antipsychotic dose compared to non-exposure (i.e., placebo or 0 mg/d). The colored areas display the 95% confidence intervals (95%CI). The color key displays the confidence in the evidence according to the GRADE approach (green=high, blue=moderate, yellow=low, red=very low). a: different formulations were pooled; b: knot locations at the 10th, 50th, and 90th percentiles were used; n. studies=number of studies; n. arms=number of arms; N=number of participants; OR odds ratio; EPS extrapyramidal side-effects.

The findings for each antipsychotic for the general population of chronically-ill patients with an acute exacerbation, and when available for other patient subgroups, are presented below and in Fig. 2. Detailed descriptions of heterogeneity assessment, sensitivity analyses, and small-study effects are presented in eAppendices 6–8. We also appraised quality of evidence for each antipsychotic drug separately using GRADE approach and reported the assessments in the results (detailed assessments are provided in eAppendix 9).

#### Amisulpride

There was low confidence of evidence of an increasing monotonic curve, i.e., higher risk of EPS with increasing doses, reaching large odds ratios (ORs) up to 5.79 95%CI [2.06, 16.25] at 1200 mg/d (number of studies *n* = 1, number of participants *N* = 255, number of arms *k* = 4; VPC not estimable; *p* value of the Wald test = 0.004). Although the single available trial did not include a placebo arm [47], ORs were calculated using 0 mg/d as the reference point. It should be noted that this is an extrapolation, which is prone to bias.

In patients with predominant negative symptoms, lower amisulpride doses up to 300 mg/d, which are sufficient for this indication [48], were investigated. There was very low confidence of evidence of a relatively flat curve with small point estimates up to a maximum of OR 1.41 [0.55, 3.60] at 80.9 mg/d, yet 95%CI could not exclude the null effect and medium-to-large ORs (*n* = 4, *N* = 591, *k* = 10; median VPC = 0%, *p* value = 0.75).

#### Aripiprazole

There was low confidence of evidence of a linear curve reaching small ORs up to 1.57 [0.97, 2.56] at 30 mg/d, but 95%CI did not exclude the null effect (*n* = 9, *N* = 2259, *k* = 26; median VPC = 52%; *p* value = 0.17).

#### Asenapine

There was moderate confidence of evidence of a linear curve reaching small-to-medium ORs up to 2.14 [1.27, 3.62] at 20 mg/d (*n* = 6, *N* = 2242, *k* = 17, median VPC = 24.2%; *p* value = 0.01).

#### Brexpiprazole

There was high confidence of evidence of an almost linear curve with small ORs reaching up to 1.68 [1.21, 2.34] at 5 mg/d (*n* = 4, *N* = 2178, *k* = 17; median VPC = 0%, *p* value = 0.006).

#### Cariprazine

There was very low confidence of evidence of a linear curve reaching up to a medium OR 3.52 [1.56, 7.96] at 9 mg/d (*n* = 5, *N* = 1918, *k* = 17; median VPC = 45.6%; *p* value = 0.01).

#### Clozapine

We found no usable data for clozapine.

#### Haloperidol

There was moderate confidence of evidence of an increasing monotonic relationship with large ORs after 10 mg/d up to a maximum OR 7.56 [3.16, 18.08] at 30 mg/day (*n* = 17, *N* = 2623, *k* = 38; median VPC = 48.2%, *p* value < 0.001).

#### Iloperidone

There was very low confidence of evidence of a relatively flat curve reaching small point estimates up to OR 1.55 [0.46, 5.26] at 24 mg/d, but 95%CI were very wide and could not exclude the null and larger effects (*n* = 2, *N* = 952, *k* = 6; median VPC = 0%, *p* value = 0.65).

#### Lumateperone

There was low confidence of evidence of a J-shaped relationship with the risk being increased above >60 mg/d reaching large point estimates up to OR 5.52 [0.38, 80.46] at 120 mg/day. However, 95%CI were very wide and could not exclude the null and larger effects (*n* = 3, *N* = 1225, *k* = 9; median VPC = 7.8%, *p* value = 0.46).

#### Lurasidone

There was low confidence of evidence of an increasing linear relationship with medium-to-large ORs for doses up to 160 mg/d, and large ORs for higher doses up to a maximum of 6.93 [3.23, 14.84] at 240 mg/d (*n* = 11, *N* = 3649, *k* = 32; median VPC = 13.6%, *p* value < 0.001). Only one study [49] investigated lurasidone dose above 160 mg/day, i.e., 240 mg/d, in treatment-resistant patients, which explained the wider 95%CI at these doses. Small-study effects were indicated by funnel plot asymmetry and dose-response meta-regression for sample size (*p* = 0.04) (eAppendix 8).

#### Olanzapine

There was moderate confidence of evidence of an almost linear relationship with negligible ORs for doses up to 20 mg/d, and small-to-medium ORs at higher doses reaching a maximum of OR 2.73 [0.78, 9.54] at 40 mg/d. Nevertheless, there were only limited data at doses >20 mg/d from a single study [50], and the 95%CI were wide and could not exclude the null and large effects (*n* = 16, *N* = 3813, *k* = 40, median VPC = 20.4%, *p* value = 0.28).

Doses up to 20 mg/d were available from one 26-weeks study in patients with predominant negative symptoms [51]. There was very low confidence of evidence of a flat curve and a maximum OR 1.14 [0.42, 3.11] at 20 mg/d, yet 95%CI were wide and did not exclude the null and medium effects (*n* = 1, *N* = 174, *k* = 3, VPC not estimable, *p* value = 0.91).

#### Paliperidone

There was high confidence of evidence of an increasing linear curve reaching small-to-medium ORs up to a maximum OR 2.07 [1.31, 3.26] at 15 mg/d (*n* = 11, *N* = 4215, *k* = 36; median VPC = 35%, *p* value = 0.007).

#### Quetiapine

There was moderate confidence of evidence of a relatively flat curve indicating no relationship and a negligible risk of a maximum OR 1.11 [0.48, 2.55] at 1200 mg/d (*n* = 9, *N* = 3058, *k* = 32; median VPC = 30.7%; *p* value = 0.94). Nevertheless, high doses >800 mg/d were only investigated in two studies with treatment-resistant patients [52, 53].

#### Risperidone

There was high confidence of evidence of an increasing linear curve with small-to-medium ORs and reaching a maximum 3.30 [2.37, 4.61] at 16 mg/d (*n* = 23, *N* = 6151, *k* = 60, median VPC = 22.4%, *p* value < 0.001).

In first-episode patients, risperidone doses up to 6 m/g were investigated. There was very low confidence of an increasing monotonic relationship reaching a maximum OR 3.53 [0.05, 232.38] at 6 mg/d. Nevertheless, the 95%CI were wide and did not exclude the null and large effects (*n* = 2, *N* = 72, *k* = 4; medium VPC = 19.4%, *p* = 0.64).

#### Sertindole

There was moderate confidence of evidence of a relatively flat curve indicating no relationship and a negligible risk up to a maximum OR 1.10 [0.70, 1.73] at 24 mg/d (*n* = 4, *N* = 1332, *k* = 15; median VPC = 0%; *p* value = 0.12).

#### Ziprasidone

There was low confidence of a linear curve with small-to-medium ORs reaching up to 3.06 [1.47, 6.38] at 320 mg/d (*n* = 8, *N* = 1785, *k* = 25, median VPC = 28.9%; *p* value = 0.003).

#### Zotepine

There was very low confidence of a relatively flat curve with small ORs reaching up to 1.38 [0.03, 68.44] at 208 mg/d, but 95%CI were very wide and could not exclude the null and larger effects (*n* = 2, *N* = 119, *k* = 4, median VPC = 40.9%; *p* value = 0.99).

### Sensitivity analyses

We only conducted sensitivity analyses in chronically-ill patients with schizophrenia, since we had sparse data in predominant negative symptoms and first-episode schizophrenia. The results did not materially change in sensitivity analyses, yet some analyses had limited power, i.e., comparisons between different formulations. A more detailed discussion is provided in eAppendix 7. Notably, there were some differences in the magnitude of odds ratios (ORs) across the different outcome measures for extrapyramidal side-effects, and particularly, ORs derived from scale-derived data tended to be smaller than from the number of patients receiving antiparkinsonian medication or those with at least one EPS (eAppendix 7). Nevertheless, their dose-response curves had generally similar shapes.

### D_2_R occupancy and risk of EPS

We *post-hoc* plotted the dose-response curves of individual antipsychotics by converting daily doses to median D_2_R occupancies using readily available formulas for aripiprazole, amisulpride, haloperidol, olanzapine paliperidone, quetiapine, risperidone, and ziprasidone [11]. We downrated the confidence in the evidence given that D_2_R occupancies were estimated from the administered doses [11]. It should also be highlighted that there were no available formulas from Lako et al 2013 to estimate D_2_R occupancies from the daily dose of the other included antipsychotics [11]. There were also no usable EPS data for clozapine, as mentioned above.

**Fig. 3:**
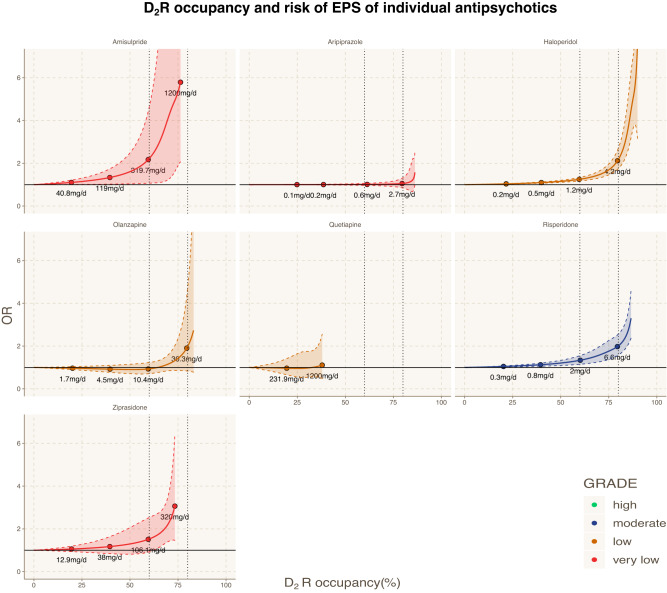
Dopamine 2 receptor (D_2_R) occupancy and risk of extrapyramidal side-effects of seven individual antipsychotics. This figure shows the relationships between dopamine 2 receptor (D_2_R) occupancies and risk of extrapyramidal side-effects (EPS) of seven individual antipsychotics (amisulpride, aripiprazole, haloperidol, olanzapine, quetiapine, risperidone, ziprasidone). The X-axis displays the median D_2_R occupancies (%) calculated from prescribed daily doses using formulas from Lako et al 2013 [11]. There were no available data and/or formulas for the other antipsychotics considered in this review. The Y-axis displays the corresponding ORs for the risk of EPS associated with a specific level of D2R occupancy compared with non-exposure (i.e., placebo or 0%). The colored areas display the 95% confidence intervals (95%CI). The color key displays the confidence in the evidence according to the GRADE approach (green=high, blue=moderate, yellow=low, red=very low). D_2_R dopamine 2 receptor; EPS extrapyramidal side-effects; OR odds ratio.

**Fig. 4:**
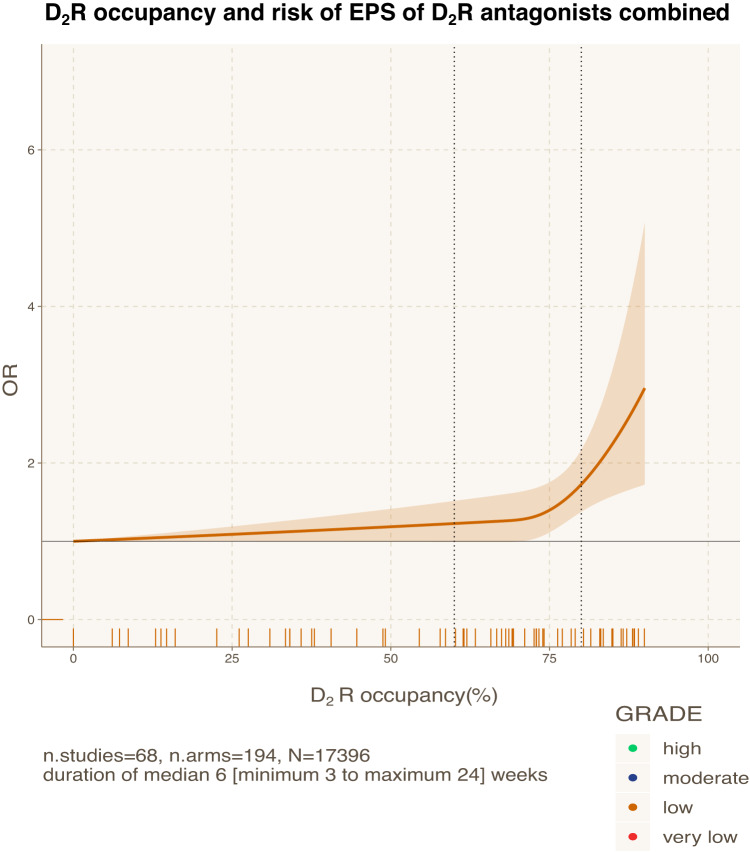
Dopamine 2 receptor (D_2_R) occupancy and risk of extrapyramidal side-effects of D_2_R antagonists combined. This figure shows the relationships between dopamine 2 receptor (D_2_R) occupancies and risk of extrapyramidal side-effects (EPS) of D_2_R antagonists combined (amisulpride, haloperidol, olanzapine, quetiapine, risperidone, ziprasidone). The X-axis displays the median D_2_R occupancies (%) calculated from prescribed daily doses using formulas from Lako et al 2013 [11]. There were no available data and/or formulas for the other antipsychotics considered in this review. The Y-axis displays the corresponding ORs for the risk of EPS associated with a specific level of D2R occupancy compared with non-exposure (i.e. placebo or 0%). The colored areas display the 95% confidence intervals (95%CI). The color key displays the confidence in the evidence according to the GRADE approach (green=high, blue=moderate, yellow=low, red=very low). D_2_R dopamine 2 receptor; EPS extrapyramidal side-effects; OR odds ratio.

The curves of D_2_R antagonists had generally a similar shape indicating a substantial increase of the risk of EPS at D_2_R occupancies above 75–85% (Fig. 3). To further explore this relationship, we combined findings for D_2_R antagonists in a dose-response meta-analysis by using the estimated median D_2_R occupancy instead of the dose (Fig. 4). Again, we found that the risk of EPS was negligible to small at D_2_R occupancies below 75–85% (e.g., OR_60%_ = 1.22 [0.99, 1.52], OR_80%_ = 1.73 [1.38, 2.17]), but increased substantially at D_2_R occupancies exceeding 75–85% reaching up to OR_90%_ = 2.96 [1.72, 5.07] (*n* = 68, *N* = 17396, *k* = 194; median VPC = 48.1%; *p* value < 0.001). However, the confidence in the evidence was low due to major concerns in indirectness, i.e., estimation of the median D_2_R occupancy from the dose of six antipsychotics (eAppendix 10).

On the other hand, the curve of the partial D_2_R agonist aripiprazole was relatively flat and the risk of EPS was small even at D_2_R occupancies above 85% (Fig. 3). As mentioned above, we could not estimate curves of the other partial D_2_R agonists, i.e., brexpiprazole and cariprazine, due to the lack of available formulas for these antipsychotics [11].

## Discussion

This dose-response meta-analysis provided the most comprehensive and up-to-date synthesis of randomized evidence on the relationship between dose, D_2_R occupancy and extrapyramidal side-effects (EPS) of antipsychotics.

### Summary of findings

We found that almost all antipsychotic drugs could cause dose-dependent EPS, except for quetiapine and sertindole (no data for clozapine, very low quality of findings for iloperidone and zotepine). However, the risk of EPS varied across antipsychotics in accordance to the well-established evidence from a previous network meta-analysis [21].

Our analysis identified two main shapes of dose-response curves: (i) monotonic or linear curves indicating a rising risk of EPS with increasing doses of haloperidol, lurasidone, amisulpride, lumateperone, cariprazine, risperidone, ziprasidone, olanzapine, asenapine, paliperidone, brexpiprazole, and aripiprazole (in descending order of their maximum ORs), and (ii) flat curves indicating no dose-response relationship for iloperidone, quetiapine, sertindole, and zotepine.

Notably, we had very low confidence in the estimates of the effects for certain groups and medications. Specifically, we had very low confidence in the estimates for individuals with predominant negative symptoms and first-episode schizophrenia, as well as for cariprazine, iloperidone, and zotepine in people with acute exacerbations. This uncertainty suggests that future research could easily change their results.

### Dose-dependent EPS and the D_2_R therapeutic window

Our findings were generally consistent with previous reviews on dose-effects [22, 54, 55] and can be at least partially explained by the D_2_R therapeutic window of antipsychotics, which suggests that risk of EPS increases when D_2_R occupancies exceed 75–85% [8, 11–14, 17].

We found not only that the risk of EPS could increase abruptly when the occupancy of D_2_R exceeds 75–85%, but also the magnitude of OR was approximately 2 at antipsychotic doses corresponding to D_2_R occupancy of 75–85% (Fig. 3). On the other hand, antipsychotics with D_2_R occupancies below 80% at their clinically effective doses, may be associated with a negligible or small risk of EPS, as we found in our analysis for quetiapine and olanzapine (recommended dose up to 20 mg/d). Similar findings have also been observed for sertindole, clozapine, and lumateperone (recommended dose 60 mg/d) [8, 56, 57], although we could not estimate the D2R occupancy for these drugs due to the lack of usable data and/or readily available formulas (eAppendix 2) [11].

Although the D_2_R therapeutic window provides a good framework for understanding the relationship between antipsychotic doses and EPS, other potential mechanisms cannot be disregarded. These mechanisms could include protective effects of slow association or fast dissociation from the D_2_R^7^, 5-HT_2A_R and 5-HT_2C_R antagonism, and 5-HT_1A_R partial agonism [8, 18–20]. In particular, nondopaminergic mechanisms like 5-HT_2A_R antagonism have been proposed to potentially explain the lower risk of EPS for certain antipsychotics such as asenapine, iloperidone, and sertindole [20]. However, this theory has been challenged, e.g., amisulpride, a relatively selective D_2_R antagonist, seems to have a comparable risk to other antipsychotics that act as 5-HT_2A_R antagonists such as risperidone [8, 21]. Nevertheless, caution is necessary when interpreting the D_2_R occupancy curve of combined antipsychotics (Fig. 4) due to their variable receptor-binding profiles [6–8, 21]. Notably, there were differences among antipsychotics at higher doses despite similar D_2_R occupancies, such as between haloperidol 11 mg/d with OR = 4.7 and risperidone 16 mg/d with OR = 3.3 even though both had D_2_R occupancy of around 87% (Fig. 3).

Additionally, partial D_2_R agonists, i.e., aripiprazole, brexpiprazole and cariprazine, do not conform to the conventional D_2_R therapeutic window due to their unique pharmacological properties [8, 42, 58, 59]. Compared to D_2_R antagonists, these compounds have a low intrinsic activity on D_2_R, which may vary depending on the brain region, receptor sensitivity and cell systems [42, 60]. At clinical effective doses, partial agonists exhibit higher D_2_R occupancies beyond 80% exerting their antipsychotic effect, while still retaining some dopaminergic signalling resulting in a reduced risk of EPS [8, 42, 58, 59].

Accordingly, we found that aripiprazole did not cause EPS at D_2_R occupancy of 80% (OR = 1.05 at 3 mg/d), and the risk remained low even at higher occupancies (up to OR = 1.57 at 30 mg/d). The other partial agonists had a higher risk compared with aripiprazole even at doses with D_2_R occupancy of about 80% [61, 62], i.e., brexpiprazole (OR = 1.42 at 4 mg/d) and cariprazine (OR = 1.69 at 3 mg/d). Their higher risk of EPS can be potentially explained by their lower intrinsic activity compared with aripiprazole [63].

### Implications to clinical practice

In our previous meta-analysis, we investigated the dose-response curves for the acute efficacy of antipsychotics and identified their near-maximal doses that achieve 95% of the maximum efficacy (ED95) [15]. Nonetheless, decisions concerning antipsychotic doses should consider potential side-effects, many of which are dose-dependent [22, 25, 64]. Hence, the present dose-response meta-analysis on extrapyramidal side-effects (EPS) can offer additional evidence-based information that can guide treatment decisions about antipsychotics for schizophrenia. To further facilitate the interpretation of the current analysis, we provided an overview of the EPS risk at the recommended and ED95 doses of antipsychotics in eAppendix 11 [15].

Most antipsychotics have a hyperbolic relationship between dose and efficacy with a plateau at ED95 doses [15], yet their dose-response curves for EPS are almost linear. For example, aripiprazole doses above ~12 mg/d, and haloperidol and risperidone doses above 6 mg/d, are not more efficacious on average [15], but can increase the risk of EPS. However, dose-effects can differ among antipsychotics. Olanzapine, for instance, may have higher efficacy at higher doses [15], but also an increased risk of EPS beyond the maximum recommended dose of 20 mg/d. Sertindole, on the other hand, may not cause EPS across doses, although higher doses may be more efficacious [15]; the risk of QTc prolongation should not be overlooked [21]. Thus, dose-response curves can optimize benefit-risk evaluations and support shared-decision-making frameworks in selecting antipsychotic medications [65].

Additionally, the linear dose-response curves for EPS observed in most antipsychotics suggest that dose reduction could potentially mitigate these adverse events, which is in line with our previous Cochrane review on antipsychotic dose reduction [66].

### Limitations

The analysis has certain limitations. First, we defined our primary outcome *post-hoc* as the number of participants receiving antiparkinsonian medications and used other proxies when data were not available. This decision was made in order to provide a comprehensive analysis and increase the power to estimate dose-response relationships. We gave preference to the use of antiparkinsonian medications as a measure of global EPS, as it was often used in previous analyses [21, 67]. We also prioritised dichotomous outcomes over scale-derived data due to skewness [67]. In a sensitivity analysis comparing dichotomous to scale-derived data (by estimating ORs from SMDs), we found that effect sizes were generally smaller for the latter (eAppendix 7). This could be potentially attributed to skewness (e.g., large SDs may dilute SMDs) and/or the transient nature of EPS during the study, resulting in small mean changes from baseline to endpoint [68]. A previous dose-response meta-analysis analysed only scale-derived data, which could also explain some of the differences in the findings with the current analysis [54].

Second, the term “EPS” is an umbrella term of heterogenous treatment-emergent movement disorders that could have different etiopathogeneses and require different treatments [1]. Our study primarily focused on parkinsonism and other movement disorders that could be treated with anticholinergic medications. Thus, the findings cannot be directly extrapolated to akathisia and tardive dyskinesia that have different pathogenetic mechanisms and need for distinct interventions [1]. For this reason, we conducted a separate dose-response meta-analysis on antipsychotic-induced akathisia, which revealed often but not always differing shapes of dose-response curves between akathisia and EPS [64]. This may also be relevant for antipsychotics like aripiprazole that have a clinically-important risk of akathisia, albeit a generally small risk of EPS [21]. In particular, we found that the risk of akathisia reached a plateau of OR = 1.8–2.0 at approximately 10–15 mg/d of aripiprazole [64], whereas the risk of EPS was trivial OR = 1.2–1.3 at these doses, but slightly increased up to OR = 1.57 at 30 mg/d (Fig. 2).

Third, we used on data from clinical trials in schizophrenia, and thus caution is needed in when extrapolating our findings to other conditions. For example, individuals with bipolar disorder may be more vulnerable to antipsychotic-induced EPS, even with aripiprazole and quetiapine [69], yet only a few clinical trials investigated fixed-doses of antipsychotics in this population [70]. In addition, there were limited data for patient subgroups that could be more sensitive to EPS, e.g., paediatric, first-episode and elderly patients.

Fourth, we analysed daily doses, which do not accurately reflect plasma concentrations, the subsequent receptor occupancy and clinical effects [10]. A high interpersonal variability can be expected, and thus, participant characteristics that could influence this relationship, e.g., age, sex, weight, pharmacogenetics, concomitant medications, and comorbidities, should be considered in the interpretation of the findings and future analysis with individual-participant-data is warranted. This limitation was taken into consideration in the estimation of the D_2_R occupancy (eAppendix 2) [11], and the confidence in the evidence was downrated accordingly (eAppendix 10).

Furthermore, the findings on D_2_R occupancy were based on only seven antipsychotics (Figs. 3 and 4) due to the lack of usable EPS data and/or formulas from Lako et al 2013 [11] to estimate the D_2_R occupancy for the other antipsychotics. Thus, we could not estimate the D_2_R occupancy for the newer compounds, such as cariprazine, brexpiprazole, lumateperone and lurasidone, as well as for potential “outlier” medications with low D_2_R occupancy risk of EPS, such as sertindole and clozapine. As a result, we had to downrate the confidence in the evidence due to indirectness (eAppendix 10).

Additionally, we a priori excluded first-generation antipsychotics except for haloperidol, which is the “gold standard” active comparator in antipsychotic trials [71]. Although first-generation antipsychotics could have been relevant to this analysis, given their generally higher risk of EPS [21], they are of limited importance in current clinical practice and have been inadequately examined, mainly in older clinical trials that often employed flexible-dosing schedules, which are ineligible for this analysis [71–73]. The reporting quality of adverse events in these earlier trials is also often inconsistent and inadequate [74–76], which may result in the available data on EPS not being presented in a way that allows for dose-response analysis.

Last, it is suggested that long-acting injections may have a lower risk of EPS [68], yet there were no clear differences in our analyses, which was however based on sparse data for the different formulations.

### Conclusion

This dose-response meta-analysis quantified the relationship between antipsychotic doses and the risk of EPS in schizophrenia. The risk of EPS increased with higher doses of antipsychotics, albeit with varying degrees across medications. Notably, quetiapine and sertindole exhibited no association with EPS even at high doses, and there were no usable data for clozapine.

The dose-response curves for antipsychotic-induced EPS, along with those for efficacy and other side-effects, can inform the decision-making about antipsychotic treatment for schizophrenia. Nevertheless, participant characteristics that could influence dose-response relationships should be considered in the interpretation of the findings and their impact should be further elucidated in future research.

Furthermore, our analysis used clinical trial data to quantify the relationship between D_2_R occupancy and the risk of EPS, and provided additional insights into the current therapeutic window for EPS.

### Supplementary information

eAppendix
