# Supplementary material for: Antipsychotic dose, dopamine D2 receptor occupancy and extrapyramidal side-effects: a systematic review and dose-response meta-analysis
Source: Mol Psychiatry. 2023 Aug 3;28(8):3267–77. doi: 10.1038/s41380-023-02203-y (PMC10618092; doi:10.1038/s41380-023-02203-y)
Supplement: Supplementary file 1 — eAppendix [file 41380_2023_2203_MOESM1_ESM.pdf]

eAppendix

Antipsychotic dose, dopamine D2 receptor occupancy and extrapyramidal side-effects: a systematic review and dose-response meta-analysis

Corresponding author:

Spyridon Siafis (spyridon.siafis@tum.de)

Table of Contents

eAppendix-1: PRISMA checklist....4

eAppendix-2: Protocol....7

PROSPERO registration....7

Difference between protocol and review....7

References....10

eAppendix-3: Search strategy and flow diagram....11

Search strategy....11

Studies identified in previous work....11

The search in the Cochrane Schizophrenia Group's Study-Based Register....11

An update search in PubMed....11

An update search in CSzG's register....12

An update search in PubMed and CENTRAL....12

PRISMA flow Diagram....14

References....14

eAppendix-4: Characteristics of included studies....16

Table of characteristics of included studies....16

Table of studies in people with an acute exacerbation of chronic schizophrenia....49

Table of studies in people with predominant negative symptoms....50

Table of studies in people with first-episode schizophrenia....50

References....50

eAppendix-5: Risk of bias assessments....58

Table of risk of bias assessments for individual studies (Cochrane risk of bias tool 1)....58

Summary of risk of bias of included studies assessed by Cochrane risk of bias tool 1....63

References....63

eAppendix-6: Heterogeneity assessments....64

Variance partition coefficients (VPC) for dose-response curves of individual antipsychotics....65

eAppendix-7: Sensitivity analyses....66

Separate dose-response meta-analyses for continuous and dichotomous outcomes of extrapyramidal side-effects....66

We conducted separate analyses for the different outcome measures contributing to the main analysis:....66

Different formulations of antipsychotics....66

Excluding studies with certain characteristics and using different knot points....67

Separate dose-response meta-analyses for continuous and dichotomous outcomes of extrapyramidal side-effects....68

Dose-response curves of different formulations of antipsychotics....69

Dose-response curves of sensitivity analyses of excluding non-dose-finding RCTs, open RCTs, studies in treatment resistant schizophrenia, and of using different knot points....70

eAppendix-8: Small-study effects assessment....71

Haloperidol *72*
Lurasidone *72*
Olanzapine *73*
Paliperidone *73*
Risperidone *74*
References *74*

eAppendix-9: Confidence in the evidence using the GRADE approach 75

Approach *75*
Risk of bias 75
Reporting bias 75
Indirectness 75
Inconsistency 75
Impression 75

Reference *76*
Confidence in the evidence for dose-response curves of individual antipsychotics *77*

eAppendix-10: Relationship between the D_2R occupancy and the risk of EPS 79

Individual antipsychotics *79*
Combined D_2R antagonists *79*
Confidence in the evidence using the GRADE approach for relationship between D_2R occupancy and risk of EPS (D_2R antagonists combined, thus not including aripiprazole) 79
VPC for the D_2R occupancy analysis for combined antipsychotics 80

References *80*

eAppendix-11: Risk of EPS at recommended and near-maximal effective doses of antipsychotics 81

Dose-response curves for EPS and recommended dose ranges of antipsychotics *82*
Table of the risk for EPS at recommended and near-maximal doses of antipsychotics *83*
References *83*

eAppendix-12: Abbreviations 84

eAppendix-1: PRISMA checklist

Section and Topic	Item #	Checklist item	Location where item is reported
**TITLE**			
Title	1	Identify the report as a systematic review.	1
**ABSTRACT**			
Abstract	2	See the PRISMA 2020 for Abstracts checklist.	2-3 (the risk of bias methods was not reported given the word count limits)
**INTRODUCTION**			
Rationale	3	Describe the rationale for the review in the context of existing knowledge.	4
Objectives	4	Provide an explicit statement of the objective(s) or question(s) the review addresses.	4
**METHODS**			
Eligibility criteria	5	Specify the inclusion and exclusion criteria for the review and how studies were grouped for the syntheses.	5-6
Information sources	6	Specify all databases, registers, websites, organisations, reference lists and other sources searched or consulted to identify studies. Specify the date when each source was last searched or consulted.	6, eAppendix-3
Search strategy	7	Present the full search strategies for all databases, registers and websites, including any filters and limits used.	eAppendix-3
Selection process	8	Specify the methods used to decide whether a study met the inclusion criteria of the review, including how many reviewers screened each record and each report retrieved, whether they worked independently, and if applicable, details of automation tools used in the process.	6
Data collection process	9	Specify the methods used to collect data from reports, including how many reviewers collected data from each report, whether they worked independently, any processes for obtaining or confirming data from study investigators, and if applicable, details of automation tools used in the process.	6
Data items	10a	List and define all outcomes for which data were sought. Specify whether all results that were compatible with each outcome domain in each study were sought (e.g. for all measures, time points, analyses), and if not, the methods used to decide which results to collect.	6-7, eAppendix-2
	10b	List and define all other variables for which data were sought (e.g. participant and intervention characteristics, funding sources). Describe any assumptions made about any missing or unclear information.	
Study risk of bias assessment	11	Specify the methods used to assess risk of bias in the included studies, including details of the tool(s) used, how many reviewers assessed each study and whether they worked independently, and if applicable, details of automation tools used in the process.	6-7, eAppendix-2
Effect measures	12	Specify for each outcome the effect measure(s) (e.g. risk ratio, mean difference) used in the synthesis or presentation of results.	7, eAppendix-2

Section and Topic	Item #	Checklist item	Location where item is reported
Synthesis methods	13a	Describe the processes used to decide which studies were eligible for each synthesis (e.g. tabulating the study intervention characteristics and comparing against the planned groups for each synthesis (item #5)).	7
	13b	Describe any methods required to prepare the data for presentation or synthesis, such as handling of missing summary statistics, or data conversions.	7, eAppendix-2
	13c	Describe any methods used to tabulate or visually display results of individual studies and syntheses.	7-8, eAppendix-2
	13d	Describe any methods used to synthesize results and provide a rationale for the choice(s). If meta-analysis was performed, describe the model(s), method(s) to identify the presence and extent of statistical heterogeneity, and software package(s) used.	7-8
	13e	Describe any methods used to explore possible causes of heterogeneity among study results (e.g. subgroup analysis, meta-regression).	8
	13f	Describe any sensitivity analyses conducted to assess robustness of the synthesized results.	8
Reporting bias assessment	14	Describe any methods used to assess risk of bias due to missing results in a synthesis (arising from reporting biases).	8-9, eAppendix-2
Certainty assessment	15	Describe any methods used to assess certainty (or confidence) in the body of evidence for an outcome.	8-9, eAppendix-2
**RESULTS**			
Study selection	16a	Describe the results of the search and selection process, from the number of records identified in the search to the number of studies included in the review, ideally using a flow diagram.	10, eAppendix-3, Figure-1
	16b	Cite studies that might appear to meet the inclusion criteria, but which were excluded, and explain why they were excluded.	n.a.
Study characteristics	17	Cite each included study and present its characteristics.	eAppendix-4
Risk of bias in studies	18	Present assessments of risk of bias for each included study.	eAppendix-5
Results of individual studies	19	For all outcomes, present, for each study: (a) summary statistics for each group (where appropriate) and (b) an effect estimate and its precision (e.g. confidence/credible interval), ideally using structured tables or plots.	Dose-responses were not conducted in the study level
Results of syntheses	20a	For each synthesis, briefly summarise the characteristics and risk of bias among contributing studies.	10
	20b	Present results of all statistical syntheses conducted. If meta-analysis was done, present for each the summary estimate and its precision (e.g. confidence/credible interval) and measures of statistical heterogeneity. If comparing groups, describe the direction of the effect.	10-16
	20c	Present results of all investigations of possible causes of heterogeneity among study results.	10-16, eAppendix-6
	20d	Present results of all sensitivity analyses conducted to assess the robustness of the synthesized results.	14, eAppendix-7
Reporting	21	Present assessments of risk of bias due to missing results (arising from reporting biases) for each synthesis assessed.	10-14, eAppendix-8,

Section and Topic	Item #	Checklist item	Location where item is reported
biases			eAppendix-10
Certainty of evidence	22	Present assessments of certainty (or confidence) in the body of evidence for each outcome assessed.	10-16, Figure-2, Figure-3, Figure-4, eAppendix-9, eAppendix-10
**DISCUSSION**			
Discussion	23a	Provide a general interpretation of the results in the context of other evidence.	16-19
	23b	Discuss any limitations of the evidence included in the review.	16-17
	23c	Discuss any limitations of the review processes used.	19-21
	23d	Discuss implications of the results for practice, policy, and future research.	18-19
**OTHER INFORMATION**			
Registration and protocol	24a	Provide registration information for the review, including register name and registration number, or state that the review was not registered.	5, eAppendix-2
	24b	Indicate where the review protocol can be accessed, or state that a protocol was not prepared.	5, eAppendix-2
	24c	Describe and explain any amendments to information provided at registration or in the protocol.	5, eAppendix-2
Support	25	Describe sources of financial or non-financial support for the review, and the role of the funders or sponsors in the review.	23
Competing interests	26	Declare any competing interests of review authors.	23
Availability of data, code and other materials	27	Report which of the following are publicly available and where they can be found: template data collection forms; data extracted from included studies; data used for all analyses; analytic code; any other materials used in the review.	n.a. Available upon reasonable request to the corresponding author

From: Page MJ, McKenzie JE, Bossuyt PM, Boutron I, Hoffmann TC, Mulrow CD, et al. The PRISMA 2020 statement: an updated guideline for reporting systematic reviews. BMJ 2021;372:n71. doi: 10.1136/bmj.n71

For more information, visit: http://www.prisma-statement.org/

eAppendix-2: Protocol

PROSPERO registration

Stefan Leucht, Tasnim Hamza, Spyridon Siafis, Hui Wu, Johannes Schneider-Thoma, John Davis. *Dose-response a meta-analysis of the efficacy and side-effects of antipsychotic drugs in schizophrenia*. PROSPERO 2020 CRD42020181467 Available from:

https://www.crd.york.ac.uk/prospero/display_record.php?ID=CRD42020181467

The registered protocol outlined the methodology of multiple publications of dose-response meta-analyses, including the current study on antipsychotic-induced extrapyramidal side-effects. Data extraction for this study began prior to the submission of the protocol to PROSPERO, as clearly indicated in the registration.

Difference between protocol and review

Primary outcome: We had planned to use the mean change from baseline to endpoint of extrapyramidal side-effects (EPS) rating scales, analysed with standardized mean difference (SMD), for the primary outcome. However, these data were heavily skewed which would yield misleading conclusions (see eAppendix-7). Furthermore, the main rating scale used in the studies, the Simpson Angus Scale (SAS), has been used in different ways, i.e., total scores were used divided or not divided by items, which made it not always possible to differentiate standard deviations from standard errors in these cases. Wrong assumptions would lead to confusing interpretations. For these reasons, we used the planned secondary outcome, number of participants who used at least once antiparkinsonian medication (often used as a proxy measure of global EPS and it was analysed with odds ratios, ORs), as the primary outcome. When data were not available, we used number of participants with at least one treatment emergent EPS (ORs) as a proxy. If both dichotomous data were not available, we analyzed mean change from baseline to endpoint of EPS scales with SMDs and transformed them to ORs, using the approach of Hasselblad and Hedges (1). To test the robustness of the results, we reported each outcome separately in the sensitivity analyses (see eAppendix-7).

Different formulations: We pooled different formulations of antipsychotics to summarize the current available data in the main analysis and tested different formulations separately in the sensitivities analyses to show whether their risks of EPS were different from each other (see eAppendix-7). In case of long-acting injections, we converted the dose to daily equivalents similar to our previous meta-analysis (2).

Sensitivity analysis for subgroups: We conducted sensitivity analyses in adults with acute exacerbation of chronic schizophrenia, but not for the other patient subgroups, i.e., predominant negative symptoms and first-episode schizophrenia, given the sparse data (see eAppendix-7).

Knot points: Knot points at the 10th, 50th and 90th were used for asenapine, because the *a priori* defined 25th, 50th and 75th quantiles could not form three unique knot points for it. A sensitivity analysis was also conducted by using knot points at 10th, 50th and 90th (eAppendix-7).

Pooled risk in the placebo group and interpretation of the odds ratios (OR): We interpretated the magnitudes of ORs, i.e., small (OR=1.52), medium (OR=2.74) and large (OR=4.72), using the method of Chen et al 2010 and assuming a control risk of 5% (3). In particular, we calculated the pooled risk in the placebo group with a random-effects meta-analysis of proportions using logit transformation (back-transformed for presentation). The pooled risk of patients on the placebo group to receive antiparkinsonian medication was 8.74% 95%CI[7.25%, 10.49%] (n=65, N=6409, I^2=76.3%), and to have at least one extrapyramidal side-effect was 5.29% 95%CI[4.17%, 6.70%] (n=73, N=7784, I^2=76.9%). Therefore, the mean pooled risk in the placebo group could be between 5-10%. We used an assumed control risk of 5% to interpret the magnitude of the ORs as reported above. Assuming a control risk of 10%, then the magnitudes of ORs could be interpreted as small (OR=1.46), medium (OR=2.50) and large (OR=4.14). The interpretation of the findings did not change whether a control risk of 5% or 10% was used.

Heterogeneity assessment: Variance partition coefficients (VPC) were used to complement the heterogeneity assessment in addition to clinical judgement and visual inspection (see eAppendix-8). VPC could be interpreted similar to the I-squared (4), and we considered substantial heterogeneity when the median VPC was >50%.

Small-study effects: We investigated small-study effects, when there were at least 10 studies available, with funnel plots of pairwise meta-analysis comparing antipsychotic (any dose combined) versus placebo and a meta-regression for sample size. We preferred this approach instead of an Egger's test (correlation between ORs and standard error) and Peter's test (weighting using number of events was not possible, given that ORs were also estimated from continuous data)(5). We also conducted a dose-response meta-regression analysis for study sample size in order to investigate small-study effects in a similar way to a previous dose-response meta-analysis (see eAppendix-9) (6).

Dopamine 2 receptor (D_2R) occupancy and risk of EPS: We *post-hoc* explored the relationship between D_2R occupancy and the ORs for EPS.
We first estimated the median D_2R occupancy from the daily dose (mg/d) of eight antipsychotics (but no available data for clozapine) using formulas presented in a previous meta-analysis of Lako et al 2013 (7). The formulas, their methodology and their limitations are presented below. There were no readily available formulas in this meta-analysis for the other antipsychotics (i.e., asenapine, brexpiprazole, cariprazine, iloperidone, lumateperone, lurasidone, paliperidone, sertindole, zotepine), which were subsequently not considered in this analysis.
Then, we plotted the dose-response curves using the D_2R occupancy in the x-axis using the above-mentioned formulas. Given that the individual drugs had a similar occupancy-curve (except for the dopamine partial agonist aripiprazole), we further explored this relationship and conducted a dose-response meta-analysis using the estimated D2R occupancy instead of the dose and combining the above-mentioned antipsychotics, except for aripiprazole. In this analysis, we set knot points at 25th, 50th, 75th of D_2R occupancies >50%, given that we expect changes at this part of the curve (8) (eAppendix-10).

Estimating the median D_2R occupancy from the daily dose using formulas provided by the meta-analysis of Lako et al 2013 (7): The previous meta-analysis of Lako et al 2013 provided precise and reliable estimates of the median D_2R occupancy from the daily doses of eight antipsychotics (see table below), which could be used in epidemiological studies investigating D_2R-related adverse events like extrapyramidal side-effects (7).

The authors used individual-participant-data from 51 molecular imaging studies investigating the relationship between D_2R occupancy and daily dose in 606 patients with schizophrenia.

They estimated the median dose-occupancy functions for each antipsychotic (see table below) by applying mixed-effects nonlinear regression and Michaelis-Menten models, i.e.:

$$D2R\ occupancy\ (\%) = OccMax * \frac{dose\ (\frac{mg}{d})}{OccD50\ + dose\ (\frac{mg}{d})}$$

The OccD50 represents the dose (in mg/d) at which 50% of the D_2R are occupied.

The OccMax represents the maximum achievable D_2R occupancy by an antipsychotic in the population. Two models are generally used to estimate the OccMax:

- Constrained models assume that at almost infinite doses of antipsychotics, all D2R can be occupied, and hence OccMax is set at 100%. While such models can be biologically plausible and have been preferred in some instances (9), the assumption of OccMax=100% may not always hold, and it has not been confirmed in human imaging studies (9, 10).
- Unconstrained models are, however, more flexible, as they can estimate the OccMax without limiting it to 100%. As a result, they have often outperformed constrained models, as observed in various studies., e.g., (7, 10). The meta-analysis by Lako et al. in 2013 also employed this approach, which led to reliable models of dose-occupancy relationships (7). However, it is important to acknowledge that there is no universally accepted gold standard method, and the accuracy of such estimations is limited to the assumptions of the underlying models.

The formulas and their limitations for each of the eight antipsychotics are presented below in the table. Lako et al 2013 found that was generally considerable interindividual variability and heterogeneity across studies (7).

Table of formulas to estimate the median D2R occupancy from the daily dose of eight antipsychotics as presented in Lako et al 2013.(7)

**Antipsychotic**	**Formula between D_2R occupancy (%) and daily dose (mg/d)**	**Comments and limitations**
Amisulpride	$D2R = \frac{85 * dose}{(dose + 137)}$	n.a.
Aripiprazole	$D2R = \frac{86.9 * dose}{(dose + 0.25)}$	The formula was based on data from one study and doses mainly between 10-30mg/d. However, smaller doses <10mg/d were also included in our review.
Clozapine	$D2R = \frac{61.7 * dose}{(dose + 125)}$	There were no available data in our analysis.
Haloperidol	$D2R = \frac{91.9 * dose}{(dose + 0.65)}$	The formula was mainly based for doses below or equal to 20mg/d. However, doses >20mg/d were also considered in our review.
Olanzapine	$D2R = \frac{96.5 * dose}{(dose + 6.46)}$	n.a.

Quetiapine	$D2R = \frac{49.1 * dose}{(dose + 352)}$	n.a.
Risperidone	$D2R = \frac{92.4 * dose}{(dose + 1.07)}$	n.a.
Ziprasidone	$D2R = \frac{82.9 * dose}{(dose + 41.7)}$	The formula was mainly based on doses 80-200mg/d. However, doses >200mg/d were also included in our review.

Confidence in the evidence: We used the GRADE approach to assess the confidence of the evidence (see eAppendix-9).

References

1. Hasselblad V, Hedges LV (1995): Meta-analysis of screening and diagnostic tests. *Psychological bulletin*. 117:167.
2. Wu H, Siafis S, Hamza T, Schneider-Thoma J, Davis JM, Salanti G, et al. (2022): Antipsychotic-Induced Weight Gain: Dose-Response Meta-Analysis of Randomized Controlled Trials. *Schizophr Bull*. 48:643-654.
3. Chen H, Cohen P, Chen S (2010): How big is a big odds ratio? Interpreting the magnitudes of odds ratios in epidemiological studies. *Communications in Statistics—simulation and Computation®*. 39:860-864.
4. Crippa A, Discacciati A, Bottai M, Spiegelman D, Orsini N (2019): One-stage dose–response meta-analysis for aggregated data. *Statistical methods in medical research*. 28:1579-1596.
5. Peters JL, Sutton AJ, Jones DR, Abrams KR, Rushton L (2006): Comparison of two methods to detect publication bias in meta-analysis. *Jama*. 295:676-680.
6. Salanti G, Peter N, Tonia T, Holloway A, White IR, Darwish L, et al. (2022): The Impact of the COVID-19 Pandemic and Associated Control Measures on the Mental Health of the General Population : A Systematic Review and Dose-Response Meta-analysis. *Ann Intern Med*.
7. Lako IM, van den Heuvel ER, Knegtering H, Bruggeman R, Taxis K (2013): Estimating dopamine D_2 receptor occupancy for doses of 8 antipsychotics: a meta-analysis. *J Clin Psychopharmacol*. 33:675-681.
8. de Greef R, Maloney A, Olsson-Gisleskog P, Schoemaker J, Panagides J (2011): Dopamine D2 occupancy as a biomarker for antipsychotics: quantifying the relationship with efficacy and extrapyramidal symptoms. *The AAPS journal*. 13:121-130.
9. Hart XM, Schmitz CN, Gründer G (2022): Molecular Imaging of Dopamine Partial Agonists in Humans: Implications for Clinical Practice. *Front Psychiatry*. 13:832209.
10. Uchida H, Takeuchi H, Graff-Guerrero A, Suzuki T, Watanabe K, Mamo DC (2011): Dopamine D2 receptor occupancy and clinical effects: a systematic review and pooled analysis. *J Clin Psychopharmacol*. 31:497-502.

eAppendix-3: Search strategy and flow diagram

Search strategy

1. Studies identified in previous work (115 studies)
2. A search in the Cochrane Schizophrenia Group's (CSzG) Study-Based Register of Trials for studies comparing two doses of SGAs/Haloperidol (inception to 9th Mar 2020) (1271 reports)
3. An update search in PubMed (9th Mar 2020 to 14th Jun 2021) (394 reports)
4. An update search in CSzG's register (17th April 2020 to 6th March 2022) (1886 reports)
5. An update search in PubMed (1st Mar 2022 to 17th Feb 2023) (975 reports) and CENTRAL (1st Mar 2022 to 20th Feb 2023) (139 reports)

Studies identified in previous work

We have done exhausted searches for the previously published meta-analyses, from which we identified 115 eligible studies, the dose-response meta-analysis of antipsychotic drugs for acute schizophrenia (1), the network meta-analysis of acute treatment for schizophrenia (2), meta-analyses in patients with first episode (3), in treatment-resistant schizophrenia (4) and predominant negative symptoms (5).
Details in the search strategies and study identification can be found in the respective reviews.
Search Results: 115 studies.

The search in the Cochrane Schizophrenia Group's Study-Based Register

A detailed description of the registry can be found in the Cochrane Schizophrenia Group's website: https://schizophrenia.cochrane.org/ and the references:(6-9)
Date: 9th March 2020
Strategy: (*Amisulpride Dosage* OR *Aripiprazole Dosage* OR *Asenapine Dosage* OR *Brexpiprazole Dosage* OR *Cariprazine Dosage* OR *Clozapine Dosage* OR *Haloperidol Decanoate Dosage* OR *Haloperidol Dosage* OR *Iloperidone Dosage* OR *Lumateperone Dosage* OR *Lurasidone Dosage* OR *Olanzapine Dosage* OR *Paliperidone Dosage* OR *Paliperidone Palmitate Dosage* OR *Quetiapine Dosage* OR *Risperidone Dosage* OR *Sertindole Dosage* OR *Ziprasidone Dosage* OR *Zotepine Dosage*) in Pairwise Comparison Field of Study Records
Search Results: There were 1249 references from 357 studies.

An update search in PubMed

Our research group has done a broader update search in PubMed, which we have used and adapted to this project
Date: 14th June 2021
Strategy: (amisulpride OR aripiprazole OR asenapine OR benperidol OR brexpiprazole OR cariprazine OR chlorpromazine OR clopenthixol OR clozapine OR flupenthixol OR fluphenazine OR fluspirilene OR haloperidol OR Iloperidone OR levomepromazine OR methotrimeprazine OR loxapine OR lumateperone OR lurasidone OR molindone OR olanzapine OR paliperidone OR penfluridol OR perazine OR perphenazine OR pimozide OR quetiapine OR sertindole OR sulpiride OR thioridazine OR thiothixene OR trifluoperazine OR ziprasidone OR zotepine OR zuclopenthixol OR risperidone) AND random* from 9.3.2020 to 14.6.2021
Search Results: 394 reports.

An update search in CSzG's register

We searched all the reports added to the register from 17 April 2020 to 06 March 2022.

Search Results: 1886 reports.

An update search in PubMed and CENTRAL

During a revision of the manuscript, we conducted an update in Pubmed and CENTRAL.
Search Results: 975 in PubMed and 139 in CENTRAL

Strategy in PubMed:

#1	(Acepromazine or Acetophenazine or Amisulpride or Aripiprazole or Asenapine or Benperidol or Blonanserin or Bromperidol or Butaperazine or Carpipramine or Chlorproethazine or Chlorpromazine or Chlorprothixene or Clocapramine or Clopenthixol or Clopentixol or Clothiapine or Clotiapine or Clozapine or Cyamemazine or Cyamepromazine or Dixyrazine or Droperidol or Fluanisone or Flupehenazine or Flupenthixol or Flupentixol or Fluphenazine or Fluspirilen or Fluspirilene or Haloperidol or Iloperidone or Levomepromazine or Levosulpiride or Lithium or Loxapine or Loxapinsuccinate or Lurasidone or Lumateperone or Melperone or Mepazine or Mesoridazine or Methotrimeprazine or Molindone or Moperone or Mosapramine or Olanzapine or Oxypertine or Paliperidone or Penfluridol or Perazine or Periciazine or Pericyazine or Perospirone or Perphenazine or Pimozide or Pipamperone or Pipothiazine or Pipotiazine or Prochlorperazine or Promazine or Promethazine or Prothipendyl or Quetiapine or Remoxipiride or Reserpine or Riospirone or Risperdal or Risperidone or Seroquel or Sertindole or Stelazine or Sulpiride or Sultopride or Thiopropazate or Thioproperazine or Thioridazine or Tiospirone or Thiothixene or Tiapride or Tiotixene or Trifluoperazine or Trifluperidol or trifluoperidol or Triflupromazine or trifluperazine or Veralipride or Ziprasidone or Zotepine or Zuclopenthixol)	186333
#2	(Antipsychoti* or Anti-psychotic* or Neurolepic* or Neurolept*)	153961
#3	"Antipsychotic Agents"[Mesh]	58729
#4	#1 OR #2 OR #3	240254
#5	((randomized controlled trial[pt]) OR (controlled clinical trial[pt]) OR (randomized[tiab]) OR (placebo[tiab]) OR (drug therapy[sh]) OR (randomly[tiab]) OR (trial[tiab]) OR (groups[tiab])) NOT (animals[mh] NOT humans[mh])	4941678
#6	"Schizophrenia"[Mesh]	114012
#7	"Paranoid Disorders"[Mesh]	4246
#8	(schizo* or hebephreni* or oligophreni* or psychotic* or psychosis or psychoses)	256664
#9	#7 OR #8 OR #9	258448
#10	#4 AND #5 AND #9	38932
#11	#10 Filters: from 2022/3/1-3000/12/12	975

Date: 17.02.2023

Strategy in CENTRAL:

#1	(Acepromazine or Acetophenazine or Amisulpride or Aripiprazole or Asenapine or Benperidol or Blonanserin or Bromperidol or Butaperazine or Carpipramine or Chlorproethazine or Chlorpromazine or Chlorprothixene or Clocapramine or Clopenthixol or Clopentixol or Clothiapine or Clotiapine or Clozapine or Cyamemazine or Cyamepromazine or Dixyrazine or Droperidol or Fluanisone or Flupehenazine or Flupenthixol or Flupentixol or Fluphenazine or Fluspirilen or Fluspirilene or Haloperidol or Iloperidone or Levomepromazine or Levosulpiride or Lithium or Loxapine or Loxapinsuccinate or Lurasidone or Lumateperone or Melperone or Mepazine or Mesoridazine or Methotrimeprazine or Molindone or Moperone or Mosapramine or Olanzapine or Oxypertine or Paliperidone or Penfluridol or Perazine or Periciazine or Pericyazine or Perospirone or Perphenazine or Pimozide or Pipamperone or Pipothiazine or Pipotiazine or Prochlorperazine or Promazine or Promethazine or Prothipendyl or Quetiapine or Remoxipiride or Reserpine or Riospirone or Risperdal or Risperidone or Seroquel or Sertindole or Stelazine or Sulpiride or Sultopride or Thiopropazate or Thioproperazine or Thioridazine or Tiospirone or Thiothixene or	20722

	Tiapride or Tiotixene or Trifluoperazine or Trifluperidol or trifluoperidol or Triflupromazine or trifluperazine or Veralipride or Ziprasidone or Zotepine or Zuclopenthixol): ti, ab, kw	
#2	(Antipsychoti* or Anti-psychotic* or Neurolepic* or Neurolept*): ti, ab, kw	328
#3	MeSH descriptor: [Antipsychotic Agents]	5600
#4	#1 OR #2 OR #3	22503
#5	MeSH descriptor: [Schizophrenia]	8975
#6	MeSH descriptor: [Paranoid Disorders]	113
#7	(schizo* or hebephreni* or oligophreni* or psychotic* or psychosis or psychoses): ti, ab, kw	12063
#8	#5 OR #6 OR #7	18145
#9	#4 AND #8	7475
#10	#9 in Trials	7286
#11	#10 with Cochrane Library publication date from March 2022 to March 2023	139

Date: 20.02.2023

PRISMA flow Diagram

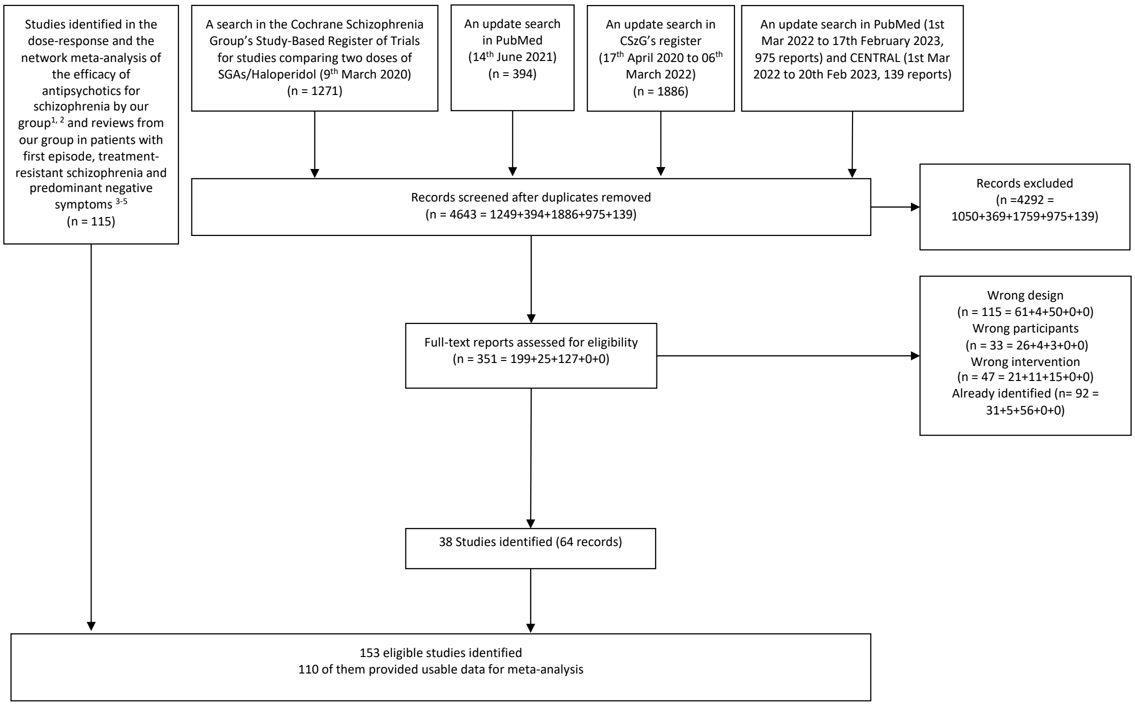

1. Leucht S, Crippa A, Siafis S, Patel MX, Orsini N, Davis JM (2020): Dose-Response Meta-Analysis of Antipsychotic Drugs for Acute Schizophrenia. *Am J Psychiatry*. 177:342-353. 2. Huhn M, Nikolakopoulou A, Schneider-Thoma J, Krause M, Samara M, Peter N, et al. (2019): Comparative efficacy and tolerability of 32 oral antipsychotics for the acute treatment of adults with multi-episode schizophrenia: a systematic review and network meta-analysis. *Lancet*. 394:939-951. 3. Zhu Y, Li C, Huhn M, Rothe P, Krause M, Bighelli I, et al. (2017): How well do patients with a first episode of schizophrenia respond to antipsychotics: a systematic review and meta-analysis. *European Neuropsychopharmacology*. 27:835-844. 4. Samara MT, Dold M, Gianatsi M, Nikolakopoulou A, Helfer B, Salanti G, et al. (2016): Efficacy, acceptability, and tolerability of antipsychotics in treatment-resistant schizophrenia: a network meta-analysis. *JAMA psychiatry*. 73:199-210. 5. Krause M, Zhu Y, Huhn M, Schneider-Thoma J, Bighelli I, Nikolakopoulou A, et al. (2018): Antipsychotic drugs for patients with schizophrenia and predominant or prominent negative symptoms: a systematic review and meta-analysis. *European archives of psychiatry and clinical neuroscience*. 268:625-639.

References

1. Leucht S, Crippa A, Siafis S, Patel MX, Orsini N, Davis JM (2020): Dose-Response Meta-Analysis of Antipsychotic Drugs for Acute Schizophrenia. *Am J Psychiatry*. 177:342-353.
2. Huhn M, Nikolakopoulou A, Schneider-Thoma J, Krause M, Samara M, Peter N, et al. (2019): Comparative efficacy and tolerability of 32 oral antipsychotics for the acute treatment of adults with multi-episode schizophrenia: a systematic review and network meta-analysis. *Lancet*. 394:939-951.
3. Zhu Y, Li C, Huhn M, Rothe P, Krause M, Bighelli I, et al. (2017): How well do patients with a first episode of schizophrenia respond to antipsychotics: a systematic review and meta-analysis. *European Neuropsychopharmacology*. 27:835-844.
4. Samara MT, Dold M, Gianatsi M, Nikolakopoulou A, Helfer B, Salanti G, et al. (2016): Efficacy, acceptability, and tolerability of antipsychotics in treatment-resistant schizophrenia: a network meta-analysis. *JAMA psychiatry*. 73:199-210.
5. Krause M, Zhu Y, Huhn M, Schneider-Thoma J, Bighelli I, Nikolakopoulou A, et al. (2018): Antipsychotic drugs for patients with schizophrenia and predominant or prominent negative symptoms: a systematic review and meta-analysis. *European archives of psychiatry and clinical neuroscience*. 268:625-639.
6. Shokraneh F, Adams CE (2021): Classification of all pharmacological interventions tested in trials relevant to people with schizophrenia: A study-based analysis. *Health Info Libr J*.
7. Shokraneh F, Adams CE (2017): Study-based registers of randomized controlled trials: Starting a systematic review with data extraction or meta-analysis. *Bioimpacts*. 7:209-217.
8. Shokraneh F, Adams CE (2019): Study-based registers reduce waste in systematic reviewing: discussion and case report. *Systematic Reviews*. 8:129.
9. Shokraneh F, Adams CE (2020): Cochrane Schizophrenia Group's Study-Based Register of Randomized Controlled Trials: Development and Content Analysis. *Schizophrenia Bulletin Open*. 1:sgaa061.

eAppendix-4: Characteristics of included studies

Table of characteristics of included studies

Study name	Intervention	Dose (mg/d)	N	Duration of illness (years)	Study duration (weeks)	Diagnosis
Arvanitis 1997(1)	Placebo	0	51	14	6	acute exacerbation of (sub-) chronic schizophrenia (DSM-III-R)
	Haloperidol	12	52	16		
	Quetiapine_IR	75	53	n.i.		
	Quetiapine_IR	150	48	n.i.		
	Quetiapine_IR	300	52	n.i.		
	Quetiapine_IR	600	51	n.i.		
	Quetiapine_IR	750	54	n.i.		
Barnas 2001(2)	Zotepine	150	6	9.4	4	acute exacerbation of schizophrenia (DSM-IV)
	Zotepine	300	6	8.7		
Beasley 1996a(3)	Placebo	0	50	14	6	schizophrenia (DSM-III-R)
	Olanzapine	1	52	16		

Study name	Intervention	Dose (mg/d)	N	Duration of illness (years)	Study duration (weeks)	Diagnosis
	Olanzapine	10	50	18		
Beasley 1996b(4)	Placebo	0	68	13	6	acute exacerbation of schizophrenia (DSM-III-R)
	Haloperidol	16.4	69	15		
	Olanzapine	6.6	65	n.i.		
	Olanzapine	11.6	64	n.i.		
	Olanzapine	16.3	69	n.i.		
Beasley 1997(5)	Haloperidol	17.6	81	12.6	6	acute exacerbation of schizophrenia (DSM-III-R)
	Olanzapine	1	88	n.i.		
	Olanzapine	6.7	87	n.i.		
	Olanzapine	11.3	86	n.i.		
	Olanzapine	16.4	89	n.i.		
Boyer 1995(6)	Placebo	0	34	9.75	6	predominant negative symptoms of schizophrenia

Study name	Intervention	Dose (mg/d)	N	Duration of illness (years)	Study duration (weeks)	Diagnosis
	Amisulpride	100	34	n.i.		(DSM-III and Andreasen's criteria for negative schizophrenia)
	Amisulpride	300	36	n.i.		
Cantillon 2014(7)	Placebo	0	39	n.i.	4	acute exacerbation of schizophrenia or schizoaffective disorder (DSM-IV-TR)
	Aripiprazole	15	20	n.i.		
Canuso 2010b(8)	Placebo	0	107	10.5	6	acute exacerbation of schizoaffective disorder (DSM-IV)
	Paliperidone	5.7	109	n.i.		
	Paliperidone	11.6	100	n.i.		
Casey 2008(9)	Placebo	0	119	n.i.	6	acute exacerbation of schizophrenia (DSM-IV-TR)
	Risperidone	6	120	n.i.		
Chouinard 1993(10)	Placebo	0	22	16	8	chronic schizophrenia (DSM-III-R)
	Haloperidol	20	21	16		
	Risperidone	2	24	n.i.		

Study name	Intervention	Dose (mg/d)	N	Duration of illness (years)	Study duration (weeks)	Diagnosis
	Risperidone	6	22	16		
	Risperidone	10	22	n.i.		
	Risperidone	16	24	n.i.		
Citrome 2021(11)	Placebo	0	206	15.7	6	acute exacerbation of schizophrenia (DSM-V)
	Asenapine_HP3070	3.8	205	15.7		
	Asenapine_HP3070	7.6	206	15.7		
Cooper 2000a(12)	Placebo	0	53	10.28	8	acute episode of schizophrenia or acute exacerbation of (sub-) chronic schizophrenia (DSM-III-R)
	Zotepine	240.57	53	10.78		
Coppola 2011(13)	Placebo	0	65	11.9	6	acute exacerbation of schizophrenia (DSM-IV)
	Paliperidone	1.5	66	16.6		
	Paliperidone	6	70	15.3		
Correll 2015(14)	Placebo	0	184	12.3	6	schizophrenia (DSM-IV-TR)

Study name	Intervention	Dose (mg/d)	N	Duration of illness (years)	Study duration (weeks)	Diagnosis
	Brexpiprazole	0.25	90	13		
	Brexpiprazole	2	182	13		
	Brexpiprazole	4	180	12.8		
Correll 2020(15)	Placebo	0	150	n.i.	4	acute exacerbation of schizophrenia (DSM-5)
	Lumateperone	40	150	n.i.		
	Lumateperone	60	150	n.i.		
Correll 2020b(16)	Placebo	0	147	14.6	12	acute exacerbation of schizophrenia (DSM-5)
	Risperidone_ISM	75[a]	145	16.1		
	Risperidone_ISM	100[a]	146	15.9		
Corrigan 2004(17)	Placebo	0	87	14.2	6	schizophrenia (DSM-IV)
	Olanzapine	15	93	12.3		
Cutler 2006(18)	Placebo	0	88	18.3	6	acute exacerbation of schizophrenia (DSM-IV)

Study name	Intervention	Dose (mg/d)	N	Duration of illness (years)	Study duration (weeks)	Diagnosis
	Aripiprazole	2	93	n.i.		
	Aripiprazole	5	92	n.i.		
	Aripiprazole	10	94	14.8		
Cutler 2008(19)	Placebo	0	152	n.i.	4	schizophrenia (DSM-IV)
	Iloperidone	24	303	n.i.		
	Ziprasidone	160	151	n.i.		
Cutler 2008a(20)	Placebo	0	117	18.3	6	acute schizophrenia (DSM-IV)
	Quetiapine_IR	800	116	16.9		
	Quetiapine_XR	400	114	18.8		
	Quetiapine_XR	600	105	18		
	Quetiapine_XR	800	113	16.3		
Daniel 1999(21)	Placebo	0	92	14.7	6	acute exacerbation of (sub-) chronic schizophrenia or schizoaffective disorder

Study name	Intervention	Dose (mg/d)	N	Duration of illness (years)	Study duration (weeks)	Diagnosis
	Ziprasidone	80	106	n.i.		(DSM-III-R)
	Ziprasidone	160	104	14.4		
Danion 1999(22)	Placebo	0	83	9.3	12	schizophrenia of residual type (DSM-III-R)
	Amisulpride	50	84	n.i.		
	Amisulpride	100	75	n.i.		
Davidson 2007(23)	Placebo	0	123	12.8	6	schizophrenia acute episode (DSM-IV)
	Olanzapine	10	128	11.9		
	Paliperidone	3	127	10.6		
	Paliperidone	9	125	11		
	Paliperidone	15	115	12.4		
Downing 2014(24)	Placebo	0	295	14.5	6	acute exacerbation of schizophrenia (DSM-IV)
	Risperidone	4	143	15.2		

Study name	Intervention	Dose (mg/d)	N	Duration of illness (years)	Study duration (weeks)	Diagnosis
Durgam 2014(25)	Placebo	0	151	11.6	6	acute exacerbation of schizophrenia (DSM-IV-TR)
	Cariprazine	1.5	145	11.4		
	Cariprazine	3	146	11.2		
	Cariprazine	4.5	147	11.1		
	Risperidone	4	140	12.3		
Durgam 2015(26)	Placebo	0	153	12.5	6	acute exacerbation of schizophrenia (DSM-IV-TR)
	Aripiprazole	10	152	12.4		
	Cariprazine	3	155	12.4		
	Cariprazine	6	157	11.7		
Durgam 2016a(27)	Placebo	0	130	17.7	6	schizophrenia (DSM-IV-TR)
	Cariprazine	3.83	128	17.2		
	Cariprazine	8.7	134	n.i.		

Study name	Intervention	Dose (mg/d)	N	Duration of illness (years)	Study duration (weeks)	Diagnosis
Egan 2013(28)	Placebo	0	83	11.2	4	schizophrenia, acutely psychotic (DSM-IV-TR)
	Olanzapine	15	47	10.7		
Fabre 1995(29)	Placebo	0	4	12	3	(sub-)chronic schizophrenia (DSM-III-R)
	Quetiapine_IR	137.5	8	12		
Garcia 2009(30)	Placebo	0	64	n.i.	6	acute exacerbation of schizophrenia (DSM-IV-TR)
	Haloperidol	10	60	n.i.		
Goff 1998(31)	Haloperidol	15	17	13.6	4	chronic or subchronic schizophrenia or schizoaffective disorder (DSM-III-R)
	Ziprasidone	4	19	n.i.		
	Ziprasidone	10	17	n.i.		
	Ziprasidone	40	17	n.i.		
	Ziprasidone	160	20	18.3		
Goff 2013(32)	Ziprasidone	160	37	16.5	8	schizophrenia or schizoaffective disorder (DSM-IV)

Study name	Intervention	Dose (mg/d)	N	Duration of illness (years)	Study duration (weeks)	Diagnosis
	Ziprasidone	320	38	13.7		
Gopal 2010(33)	Placebo	0	136	15	13	schizophrenia (DSM-IV)
	Paliperidone palmitate (one-monthly injection)	50[a]	94	14		
	Paliperidone palmitate (once-monthly injection)	100[a]	97	14		
	Paliperidone palmitate (once-monthly injection)	150[a]	30	17		
Hale 2000(34)	Haloperidol	10	125	9.5	8	schizophrenia (DSM-III-R)
	Sertindole	8	120	n.i.		
	Sertindole	16	127	n.i.		
	Sertindole	20	128	n.i.		
	Sertindole	24	117	n.i.		
Heinrich 1994(35)	Clozapine	400	20	n.i.	4	acute schizophrenia or schizoaffective disorder (ICD-9)
	Risperidone	4	20	n.i.		

Study name	Intervention	Dose (mg/d)	N	Duration of illness (years)	Study duration (weeks)	Diagnosis
	Risperidone	8	20	n.i.		
Hera 041-021(36)	Placebo	0	106	n.i.	6	acute exacerbation of schizophrenia (DSM-IV)
	Asenapine	10	106	n.i.		
	Asenapine	20	102	n.i.		
	Olanzapine	15	103	n.i.		
Higuchi 2019a(37)	Placebo	0	152	15.36	6	schizophrenia (DSM-IV-TR)
	Lurasidone	40	150	13.95		
	Lurasidone	80	155	15.68		
Higuchi 2019b(38)	Placebo	0	133	n.i.	6	schizophrenia (DSM-IV)
	Lurasidone	40	131	n.i.		
	Lurasidone	80	131	n.i.		
	Risperidone	4	65	n.i.		

Study name	Intervention	Dose (mg/d)	N	Duration of illness (years)	Study duration (weeks)	Diagnosis
Hirayasu 2010(39)	Placebo	0	138	17.92	6	schizophrenia with acute symptoms (DSM-IV)
	Olanzapine	10	47	17.92		
	Paliperidone	6	136	17.92		
Honer 2010(40)	Quetiapine_IR	799	43	n.i.	8	schizophrenia or schizoaffective disorder (DSM-IV)
	Quetiapine_IR	1144	88	n.i.		
Ishigooka 2018(41)	Placebo	0	116	17.4	6	schizophrenia (DSM-IV-TR)
	Brexpiprazole	1	115	17.1		
	Brexpiprazole	2	115	14.6		
	Brexpiprazole	4	113	16.8		
Iyo 2021(42)	Placebo	0	236	10.4	6	acute exacerbation of schizophrenia (DSM-IV-TR)
	Lurasidone	40	247	10.6		
Kahn 2007(43)	Placebo	0	118	8	6	acute schizophrenia (DSM-IV)

Study name	Intervention	Dose (mg/d)	N	Duration of illness (years)	Study duration (weeks)	Diagnosis
	Quetiapine_IR	400	123	n.i.		
	Quetiapine_XR	400	113	n.i.		
	Quetiapine_XR	600	113	n.i.		
	Quetiapine_XR	800	121	n.i.		
Kane 2002(44)	Placebo	0	106	16	4	schizophrenia or schizoaffective disorder, acute relapse (DSM-IV)
	Aripiprazole	15	102	n.i.		
	Aripiprazole	30	102	n.i.		
	Haloperidol	10	104	16		
Kane 2003(45)	Placebo	0	98	n.i.	12	schizophrenia (DSM-IV)
	Risperidone_Consta	25[b]	99	n.i.		
	Risperidone_Consta	50[b]	103	n.i.		
	Risperidone_Consta	75[b]	100	n.i.		

Study name	Intervention	Dose (mg/d)	N	Duration of illness (years)	Study duration (weeks)	Diagnosis
Kane 2007b(46)	Placebo	0	127	9.9	6	acute episode of schizophrenia (DSM-IV)
	Olanzapine	10	128	9.8		
	Paliperidone	6	123	n.i.		
	Paliperidone	9	122	n.i.		
	Paliperidone	12	130	n.i.		
Kane 2010a(47)	Placebo	0	123	12.5	6	acute exacerbation of schizophrenia (DSM-IV-TR)
	Asenapine	10	114	n.i.		
	Asenapine	20	106	n.i.		
	Haloperidol	8	115	12.5		
Kane 2014(48)	Placebo	0	172	n.i.	12	acute exacerbation of schizophrenia (DSM-IV-TR)
	Aripiprazole_Maintena	396.4[a]	168	n.i.		
Kane 2015a(49)	Placebo	0	184	13.7	6	schizophrenia (DSM-IV-TR)

Study name	Intervention	Dose (mg/d)	N	Duration of illness (years)	Study duration (weeks)	Diagnosis
	Brexpiprazole	1	120	12.8		
	Brexpiprazole	2	186	11.7		
	Brexpiprazole	4	184	13.1		
Kane 2015b(50)	Placebo	0	147	11	6	schizophrenia (DSM-IV-TR)
	Cariprazine	5.58	151	11.3		
	Cariprazine	8.39	148	9.9		
Keck 1998(51)	Placebo	0	48	17.3	4	acute exacerbation of subchronic or chronic schizophrenia or schizoaffective disorder (DSM-III-R)
	Ziprasidone	40	44	n.i.		
	Ziprasidone	120	47	14.6		
King 1998(52)	Quetiapine_IR	50	209	11	6	acute exacerbation of chronic or subchronic schizophrenia (DSM-IIIR)
	Quetiapine_IR	450	200	10		
	Quetiapine_IR	450	209	10		

Study name	Intervention	Dose (mg/d)	N	Duration of illness (years)	Study duration (weeks)	Diagnosis
Kinon 2006c(53)	Olanzapine	10	199	17.96	8	schizophrenia or schizoaffective disorder (DSM-IV)
	Olanzapine	20	200	17.13		
	Olanzapine	40	200	17.63		
Kinon 2011(54)	Placebo	0	122	12.5	4	schizophrenia (DSM-IV)
	Olanzapine	15	62	15		
Kinoshita 2016(55)	Placebo	0	174	n.i.	6	schizophrenia (DSM-IV-TR)
	Asenapine	10	176	n.i.		
	Asenapine	20	182	n.i.		
Kramer 2010(56)	Placebo	0	84	12	9	schizophrenia (DSM-IV)
	Paliperidone palmitate (once-monthly injection)	50[a]	79	13		
	Paliperidone palmitate (once-monthly injection)	100[a]	84	12		
Landbloom 2016(57)	Placebo	0	103	n.i.	6	schizophrenia of paranoid, disorganized, or undifferentiated subtype

Study name	Intervention	Dose (mg/d)	N	Duration of illness (years)	Study duration (weeks)	Diagnosis
	Asenapine	5	98	n.i.		(DSM-IV-TR)
	Asenapine	10	113	n.i.		
	Olanzapine	15	46	n.i.		
Lane 2001(58)	Risperidone	3	11	n.i.	6	first-episode schizophrenia (DSM-IV)
	Risperidone	6	12	n.i.		
Lauriello 2008(59)	Placebo	0	98	18.8	8	schizophrenia (DSM-IV or DSM-IV-TR)
	Olanzapine_LAI	405[a]	100	16.7		
	Olanzapine_LAI	210[b]	106	16.3		
	Olanzapine_LAI	300[b]	100	18		
Lecrubier 2006(60)	Placebo	0	34	15.42	26	schizophrenia, residual, disorganised or catatonic (DSM-IV)
	Amisulpride	150	70	12.33		
	Olanzapine	5	70	n.i.		

Study name	Intervention	Dose (mg/d)	N	Duration of illness (years)	Study duration (weeks)	Diagnosis
	Olanzapine	20	70	11.08		
Liebermann 2015(61)	Placebo	0	85	16.7	4	schizophrenia (DSM-IV)
	Lumateperone	60	84	16		
	Lumateperone	120	84	n.i.		
	Risperidone	4	82	15.2		
Lindenmayer 2008(62)	Placebo	0	84	14.7	6	schizophrenia, acute exacerbation (DSM-IV)
	Quetiapine_IR	300	90	n.i.		
	Quetiapine_IR	600	86	n.i.		
	Quetiapine_XR	300	91	n.i.		
	Quetiapine_XR	600	92	n.i.		
	Quetiapine_XR	800	89	n.i.		
Lindenmayer 2011(63)	Quetiapine_IR	600	31	n.i.	8	schizophrenia or schizoaffective disorder with suboptimal treatment response (DSM-IV-R)

Study name	Intervention	Dose (mg/d)	N	Duration of illness (years)	Study duration (weeks)	Diagnosis
	Quetiapine_IR	1200	29	n.i.		
Litman 2016(64)	Placebo	0	55	n.i.	4	schizophrenia (DSM-IV)
	Risperidone	4	31	n.i.		
Loebel 2013(65)	Placebo	0	122	11.3	6	acute exacerbation of schizophrenia (DSM-IV-TR)
	Lurasidone	80	125	n.i.		
	Lurasidone	160	121	n.i.		
	Quetiapine_XR	600	120	12.4		
Loebel 2015a(66)	Placebo	0	112	14.2	6	acute exacerbation of schizophrenia (DSM-IV-TR)
	Lurasidone	20	101	14.9		
	Lurasidone	97.37	199	14.1		
Loo 1997(67)	Placebo	0	72	10.7	26	schizophrenia with predominant negative symptoms (DSM-III-R)
	Amisulpride	100	69	9.7		

Study name	Intervention	Dose (mg/d)	N	Duration of illness (years)	Study duration (weeks)	Diagnosis
Marder 1994(68)	Placebo	0	66	15.2	8	schizophrenia (DSM-III-R)
	Haloperidol	20	66	15.4		
	Risperidone	2	63	n.i.		
	Risperidone	6	64	15.8		
	Risperidone	10	65	n.i.		
	Risperidone	16	64	n.i.		
Marder 2007c(69)	Placebo	0	110	16	6	acute exacerbation of schizophrenia (DSM-IV)
	Olanzapine	10	110	15.95		
	Paliperidone	6	112	16.7		
	Paliperidone	12	112	17		
McEvoy 2007b(70)	Placebo	0	108	16.7	6	acute exacerbation of schizophrenia (DSM-IV)
	Aripiprazole	10	106	n.i.		

Study name	Intervention	Dose (mg/d)	N	Duration of illness (years)	Study duration (weeks)	Diagnosis
	Aripiprazole	15	106	n.i.		
	Aripiprazole	20	100	n.i.		
Meltzer 2004(71)	Placebo	0	98	n.i.	6	acute schizophrenia or schizoaffective disorder (DSM-IV)
	Haloperidol	10	98	n.i.		
Meltzer 2007a(72)	Placebo	0	149	n.i.	6	acute exacerbation of schizophrenia (DSM-IV-TR)
	Risperidone	6	154	n.i.		
Meltzer 2011(73)	Placebo	0	116	12.6	6	acute exacerbation of schizophrenia (DSM-IV)
	Lurasidone	40	120	n.i.		
	Lurasidone	120	119	n.i.		
	Olanzapine	15	123	13.2		
Meltzer 2012(74)	Haloperidol	2	87	n.i.	6	schizophrenia with a recent acute exacerbation of psychotic symptoms (DSM-IV)
	Risperidone	2	86	n.i.		

Study name	Intervention	Dose (mg/d)	N	Duration of illness (years)	Study duration (weeks)	Diagnosis
	Risperidone	6	87	n.i.		
Meltzer 2014(75)	Risperidone_Consta	50[b]	82	17.9	24	treatment resistant schizophrenia or schizoaffective disorder (DSM-IV)
	Risperidone_Consta	100[b]	78	20		
Meltzer 2020(76)	Lurasidone	80	34	27.7	24	treatment-resistant schizophrenia or schizoaffective disorder (DSM-IV-TR)
	Lurasidone	240	33	24.8		
Merlo 2000(77)	Risperidone	2	23	n.i.	8	first episode schizophrenia, schizophreniform disorder, or schizoaffective disorder (DSM-IV)
	Risperidone	4	26	n.i.		
Nakamura 2009(78)	Placebo	0	90	n.i.	6	schizophreniform or schizoaffective disorder or catatonic or residual subtypes of schizophrenia (DSM-IV)
	Lurasidone	80	90	n.i.		
Nakamura 2016(79)	Cariprazine	3	11	15.4	12	schizophrenia (DSM-IV-TR)
	Cariprazine	6	16	15.5		

Study name	Intervention	Dose (mg/d)	N	Duration of illness (years)	Study duration (weeks)	Diagnosis
	Cariprazine	9	11	14.6		
Nasrallah 2010(80)	Placebo	0	127	n.i.	13	schizophrenia (DSM-IV-TR)
	Paliperidone palmitate (once-monthly injection)	25[a]	131	n.i.		
	Paliperidone palmitate (once-monthly injection)	50[a]	129	n.i.		
	Paliperidone palmitate (once-monthly injection)	100[a]	131	n.i.		
Nasrallah 2013(81)	Placebo	0	128	14	6	schizophrenia (DSM-IV)
	Lurasidone	40	125	16.4		
	Lurasidone	80	123	13.5		
	Lurasidone	120	124	13.2		
Nasser 2016(82)	Placebo	0	119	n.i.	8	acute exacerbation of schizophrenia (DSM-IV-TR)
	Risperidone_RBP-7000	90[a]	116	n.i.		
	Risperidone_RBP-7000	120[a]	119	n.i.		

Study name	Intervention	Dose (mg/d)	N	Duration of illness (years)	Study duration (weeks)	Diagnosis
NCT00563706(83)	Placebo	0	37	n.i.	4	schizophrenia (DSM-IV-TR)
	Risperidone	4	43	n.i.		
NCT00905307(84)	Placebo	0	95	n.i.	6	schizophrenia (DSM-IV-TR)
	Aripiprazole	15	50	n.i.		
	Brexpiprazole	0.25	42	n.i.		
	Brexpiprazole	1	89	n.i.		
	Brexpiprazole	2.5	90	n.i.		
	Brexpiprazole	5	93	n.i.		
NCT01625000(85)	Placebo	0	126	n.i.	6	acute exacerbation of schizophrenia (DSM-IV-TR)
	Cariprazine	3	126	n.i.		
	Cariprazine	6	133	n.i.		
	Cariprazine	9	67	n.i.		

Study name	Intervention	Dose (mg/d)	N	Duration of illness (years)	Study duration (weeks)	Diagnosis
	Risperidone	4	56	n.i.		
NCT02469155(86)	Placebo	0	174	n.i.	6	acute exacerbation of schizophrenia (DSM-V)
	Lumateperone	20	174	n.i.		
	Lumateperone	60	174	n.i.		
	Risperidone	4	174	n.i.		
Ogasa 2012(87)	Placebo	0	50	n.i.	6	schizophrenia (DSM-IV)
	Lurasidone	40	50	n.i.		
	Lurasidone	120	49	n.i.		
Oosthuizen 2004(88)	Haloperidol	2	20	n.i.	6	first episode schizophreniform disorder, schizophrenia or schizoaffective disorder (DSM-IV)
	Haloperidol	8	20	n.i.		
Pandina 2010(89)	Placebo	0	164	n.i.	13	schizophrenia (DSM-IV)
	Paliperidone palmitate (once-monthly injection)	25[a]	160	n.i.		

Study name	Intervention	Dose (mg/d)	N	Duration of illness (years)	Study duration (weeks)	Diagnosis
	Paliperidone palmitate (once-monthly injection)	100[a]	165	n.i.		
	Paliperidone palmitate (once-monthly injection)	150[a]	163	n.i.		
Patil 2007(90)	Placebo	0	63	n.i.	4	schizophrenia (DSM-IV-TR)
	Olanzapine	15	34	n.i.		
Peuskens 1995(91)	Haloperidol	10	226	16.1	8	chronic schizophrenia (DSM-III-R)
	Risperidone	1	229	n.i.		
	Risperidone	4	227	n.i.		
	Risperidone	8	230	n.i.		
	Risperidone	12	226	n.i.		
	Risperidone	16	224	n.i.		
Potkin 2003(92)	Placebo	0	103	n.i.	4	schizophrenia or schizoaffective disorder (DSM-IV)
	Aripiprazole	20	101	n.i.		

Study name	Intervention	Dose (mg/d)	N	Duration of illness (years)	Study duration (weeks)	Diagnosis
	Aripiprazole	30	101	n.i.		
	Risperidone	6	99	n.i.		
Potkin 2007c(93)	Placebo	0	62	n.i.	6	acute exacerbation of schizophrenia (DSM-IV)
	Asenapine	10	60	n.i.		
	Risperidone	6	60	n.i.		
Potkin 2008a(94)	Placebo	0	127	15.6	6	acute exacerbation of schizophrenia (DSM-IV)
	Haloperidol	15	124	15.6		
	Iloperidone	4	121	n.i.		
	Iloperidone	8	125	n.i.		
	Iloperidone	12	124	15.6		
Potkin 2015(95)	Placebo	0	72	n.i.	6	acute exacerbation of schizophrenia (DSM-IV)
	Haloperidol	10	73	n.i.		

Study name	Intervention	Dose (mg/d)	N	Duration of illness (years)	Study duration (weeks)	Diagnosis
	Lurasidone	20	71	n.i.		
	Lurasidone	40	69	n.i.		
	Lurasidone	80	71	n.i.		
Puech 1998(96)	Amisulpride	100	61	n.i.	4	chronic or subchronic schizophrenia with acute exacerbation (DSM-III-R)
	Amisulpride	400	64	n.i.		
	Amisulpride	800	65	n.i.		
	Amisulpride	1200	65	n.i.		
	Haloperidol	16	64	10.7		
Santos 1989(97)	Haloperidol	10	10	2.806666667	3	schizophrenia (DSM-III)
	Haloperidol	15	10	2.806666667		
	Haloperidol	30	10	2.806666667		
Schmidt 2014(98)	Placebo	0	101	10.9	6	acute exacerbation of schizophrenia (DSM-IV)

Study name	Intervention	Dose (mg/d)	N	Duration of illness (years)	Study duration (weeks)	Diagnosis
	Olanzapine	15	93	10.7		
Simpson 1967(99)	Placebo	0	8	n.i.	14	schizophrenia (clinical diagnosis)
	Haloperidol	6	8	n.i.		
	Haloperidol	30	8	n.i.		
Study 104(100)	Placebo	0	50	n.i.	4	acute exacerbation of schizophrenia or schizoaffective disorder (DSM-III-R)
	Ziprasidone	10	47	n.i.		
	Ziprasidone	40	55	n.i.		
	Ziprasidone	80	48	n.i.		
Study 115 2000(101)	Placebo	0	83	n.i.	6	acute exacerbation of schizophrenia or schizoaffective disorder (DSM-III-R)
	Haloperidol	15	85	n.i.		
	Ziprasidone	40	87	n.i.		
	Ziprasidone	120	78	n.i.		

Study name	Intervention	Dose (mg/d)	N	Duration of illness (years)	Study duration (weeks)	Diagnosis
	Ziprasidone	200	86	n.i.		
Study 128-301 1997(102)	Haloperidol	10	120	n.i.	12	schizophrenia or schizoaffective disorder (DSM-III-R)
	Haloperidol	20	118	n.i.		
	Ziprasidone	40	116	n.i.		
	Ziprasidone	120	115	n.i.		
	Ziprasidone	200	128	n.i.		
Study 93202 2002(103)	Placebo	0	35	n.i.	4	schizophrenia acute relapse (DSM-III-R)
	Aripiprazole	30	34	n.i.		
	Haloperidol	20	34	n.i.		
Study 94202 2002(103)	Placebo	0	64	n.i.	4	schizophrenia acute relapse (DSM-IV)
	Aripiprazole	2	59	n.i.		
	Aripiprazole	10	60	n.i.		

Study name	Intervention	Dose (mg/d)	N	Duration of illness (years)	Study duration (weeks)	Diagnosis
	Aripiprazole	30	61	n.i.		
	Haloperidol	10	63	n.i.		
Study RIS-USA-72 1996(104)	Placebo	0	83	n.i.	4	schizophrenia (DSM-IV)
	Risperidone	4	85	n.i.		
	Risperidone	8	78	n.i.		
Takahashi 2013(105)	Placebo	0	164	16.5	13	schizophrenia (DSM-IV-TR)
	Paliperidone palmitate (once-monthly injection)	75[a]	160	18.6		
van Kammen 1996(106)	Placebo	0	48	13.8	5.7	schizophrenia (DSM-III-R)
	Sertindole	8	52	n.i.		
	Sertindole	12	51	n.i.		
	Sertindole	20	54	n.i.		
Walling 2019(107)	Placebo	0	74	n.i.	4	schizophrenia (DSM-IV-TR)

Study name	Intervention	Dose (mg/d)	N	Duration of illness (years)	Study duration (weeks)	Diagnosis
	Risperidone	6	37	n.i.		
Zborowski 1995(108)	Placebo	0	116	15.4	8	schizophrenia (DSM-III-R/DSM-IV)
	Haloperidol	16	115	15.4		
	Sertindole	20	117	15.7		
	Sertindole	24	113	13.8		
Zimbroff 1997(109)	Placebo	0	73	16.6	8	schizophrenia (DSM-III-R/DSM-IV)
	Haloperidol	4	71	n.i.		
	Haloperidol	8	67	n.i.		
	Haloperidol	16	70	n.i.		
	Sertindole	12	76	n.i.		
	Sertindole	20	68	n.i.		
	Sertindole	24	72	n.i.		

N= number of participants randomised, ICD 9/10 = International Classification of Diseases, 9th/10th Revision, DSM-III, -III-R, -IV, -IV-TR, -V = different versions of the Diagnostic and Statistical Manual of Mental Disorders, n.i. = not indicated, IR= immediate release, XR= extended release, LAI= long-acting injectable, a: once-monthly injection, b: fortnightly

injection. Some reports provided data for several studies.

Table of studies in people with an acute exacerbation of chronic schizophrenia

Antipsychotics	Studies
Amisulpride	Puech 1998
Aripiprazole	Cantillon 2014, Cutler 2006, Kane 2002, Kane 2014, McEvoy 2007b, NCT00905307, Potkin 2003, Study 93202 2002, Study 94202 2002
Asenapine	Citrome 2021, Hera 041-021, Kane 2010a, Kinoshita 2016, Landbloom 2016, Potkin 2007c
Brexpiprazole	Correll 2015, Ishigooka 2018, Kane 2015a, NCT00905307
Cariprazine	Durgam 2014, Durgam 2016a, Kane 2015b, Nakamura 2016, NCT01625000
Clozapine	No study included
Haloperidol	Arvanitis 1997, Beasley 1996b, Chouinard 1993, Kane 2002, Kane 2010a, Marder 1994, Meltzer 2004, Potkin 2008a, Potkin 2015, Santos 1989, Simpson 1967, Study 115 2000, Study 128-301 1997, Study 93202 2002, Study 94202 2002, Zborowski 1995, Zimbroff 1997
Iloperidone	Cutler 2008, Potkin 2008a*
Lumateperone	Correll 2020, Liebermann 2015, NCT02469155
Lurasidone	Higuchi 2019a, Higuchi 2019b, Iyo 2021, Loebel 2013, Loebel 2015a, Meltzer 2011, Meltzer 2020, Nakamura 2009, Nasrallah 2013, Ogasa 2012, Potkin 2015
Olanzapine	Beasley 1996a, Beasley 1996b, Beasley 1997, Corrigan 2004, Egan 2013, Hera 041-021, Hirayasu 2010, Kane 2007b, Kinon 2006c, Kinon 2011, Landbloom 2016, Lauriello 2008, Marder 2007c, Meltzer 2011, Patil 2007, Schmidt 2014
Paliperidone	Canuso 2010b, Coppola 2011, Davidson 2007, Gopal 2010, Hirayasu 2010, Kane 2007b, Nasrallah 2010, Pandina 2010, Takahashi 2013
Quetiapine	Arvanitis 1997, Cutler 2008a, Fabre 1995, Honer 2010, Kahn 2007, King 1998, Lindenmayer 2008, Lindenmayer 2011, Loebel 2013
Risperidone	Casey 2008, Chouinard 1993, Correll 2020b, Downing 2014, Durgam 2014, Heinrich 1994, Higuchi 2019b, Kane 2003, Liebermann 2015, Litman 2016, Marder 1994, Meltzer 2007a, Meltzer 2012, Nasser 2016, NCT00563706, NCT01625000, NCT02469155, Peuskens 1995, Potkin 2003, Potkin 2007c, Study RIS-USA-72 1996, Walling 2019
Sertindole	Hale 2000, Van Kammen 1996, Zborowski 1995, Zimbroff 1997
Ziprasidone	Cutler 2008, Daniel 1999, Goff 1998, Goff 2013, Keck 1998, Study 104, Study 115 2000, Study 128-301 1997
Zotepine	Cooper 2000a, Barnas 2001

*****For Potkin 2008a, we used the pooled risk from three studies as reported from one of the records of this study in order to estimate the number of events in iloperidone doses and placebo.

Table of studies in people with predominant negative symptoms

Antipsychotics	Studies
Amisulpride	Boyer 1995, Danion 1999, Lecrubier 2006, Loo 1997
Olanzapine	Lecrubier 2006

Table of studies in people with first-episode schizophrenia

Antipsychotics	Studies
Risperidone	Lane 2001, Merlo 2000

References

1. Arvanitis LA, Miller BG, group Sts (1997): Multiple fixed doses of "Seroquel" (quetiapine) in patients with acute exacerbation of schizophrenia: a comparison with haloperidol and placebo. *BiolPsychiatry*. 42:233-246.
2. Barnas C, Quiner S, Tauscher J, Hilger E, Willeit M, Küfferle B, et al. (2001): In vivo 123 I IBZM SPECT imaging of striatal dopamine 2 receptor occupancy in schizophrenic patients. *Psychopharmacology*. 157:236-242.
3. Beasley CM, Sanger T, Satterlee W (1996): Olanzapine versus placebo: results of a double-blind fixed dose olanzapine trial. *Psychopharmacology*. 124:159-167.
4. Beasley CM, Tollefson GD, Tran P, Satterlee W, Sanger T, Hamilton S, et al. (1996): Olanzapine versus haloperidol and placebo. Acute phase results of the american double-blind olanzapine trial. *Neuropsychopharmacology*. 14:111-123.
5. Beasley CM, Hamilton SH, Crawford AM, Dellva MA, Tollefson GD, Tran PV, et al. (1997): Olanzapine versus haloperidol: acute phase results of the international double-blind olanzapine trial. *EurNeuropsychopharmacol*. 7:125-137.
6. Boyer P, Lecrubier Y, Puech AJ, Dewailly J, Aubin F (1995): Treatment of negative symptoms in schizophrenia with amisulpride. *British Journal of Psychiatry*, pp 68-72.
7. Cantillon M (2014): Efficacy and safety of novel dopamine serotonin stabilizer rp 5063 in acute schizophrenia and schizoaffective disorder. *Schizophrenia Research*.S22.
8. Canuso CM, Lindenmayer JP, Kosik-Gonzalez C, Turkoz I, Carothers J, Bossie CA, et al. (2010): A randomized, double-blind, placebo-controlled study of 2 dose ranges of paliperidone extended-release in the treatment of subjects with schizoaffective disorder. *J Clin Psychiatry*. 71:587-598.
9. Casey DE, Sands EE, Heisterberg J, Yang H-M (2008): Efficacy and safety of bifeprunox in patients with an acute exacerbation of schizophrenia: results from a randomized, double-blind, placebo-controlled, multicenter, dose-finding study. *Psychopharmacology*. 200:317-331.
10. Chouinard G, Jones B, Remington G (1993): Canadian placebo-controlled study of fixed doses of risperidone and haloperidol in the treatment of chronic schizophrenic patients. *JClinPsychopharmacol*. 13:25-40.
11. NCT02876900 Study to Assess Efficacy and Safety of HP3070 in Subjects Diagnosed With Schizophrenia. https://ClinicalTrials.gov/show/NCT02876900.

12. Cooper S, Tweed J, Raniwalla J, Butler A, Welch C (2000): A placebo-controlled comparison of zotepine versus chlorpromazine in patients with acute exacerbation of schizophrenia. *Acta Psychiatrica Scandinavica*. 101:218-225.
13. Coppola D, Melkote R, Lannie C, Singh J, Nuamah I, Gopal S, et al. (2011): Efficacy and Safety of Paliperidone Extended Release 1.5 mg/day—A Double-blind, Placebo-and Active-Controlled, Study in the Treatment of Patients with Schizophrenia. *Psychopharmacology bulletin*. 44:54.
14. Correll CU, Skuban A, Ouyang J, Hobart M, Pfister S, McQuade RD, et al. (2015): Efficacy and Safety of Brexpiprazole for the Treatment of Acute Schizophrenia: A 6-Week Randomized, Double-Blind, Placebo-Controlled Trial. *Am J Psychiatry*. 172:870-880.
15. Correll CU, Davis RE, Weingart M, Saillard J, O'Gorman C, Kane JM, et al. (2020): Efficacy and Safety of Lumateperone for Treatment of Schizophrenia: A Randomized Clinical Trial. *JAMA Psychiatry*.
16. Correll CU, Litman RE, Filts Y, Llaudó J, Naber D, Torres F, et al. (2020): Efficacy and safety of once-monthly Risperidone ISM(®) in schizophrenic patients with an acute exacerbation. *NPJ Schizophr*. 6:37.
17. Corrigan MH, Gallen CC, Bonura ML, Merchant KM, Group SS (2004): Effectiveness of the selective D4 antagonist sonepiprazole in schizophrenia: a placebo-controlled trial. *Biological psychiatry*. 55:445-451.
18. Cutler AJ, Marcus RN, Hardy SA, O'Donnell A, Carson WH, McQuade RD (2006): The efficacy and safety of lower doses of aripiprazole for the treatment of patients with acute exacerbation of schizophrenia. *CNSSpectr*. 11:691-702.
19. Cutler AJ, Kalali AH, Weiden PJ, Hamilton J, Wolfgang CD (2008): Four-week, double-blind, placebo-and ziprasidone-controlled trial of iloperidone in patients with acute exacerbations of schizophrenia. *Journal of clinical psychopharmacology*. 28:S20-S28.
20. Cutler AJ, Tran-Johnson T, Kalali A, Astrom M, Brecher M, Meulien D (2010): A failed 6-week,randomized, double-blind, placebo-controlled study of once-daily extended release quetiapine fumarate in patients with acute schizophrenia: lessons learned. *Psychopharmacol Bull*. 43:37-69.
21. Daniel DG, Zimbroff DL, Potkin SG, Reeves KR, Harrigan EP, Lakshminarayanan M (1999): Ziprasidone 80 mg/day and 160 mg/day in the acute exacerbation of schizophrenia and schizoaffective disorder: A 6-week placebo-controlled trial. *Neuropsychopharmacology*. 20:491-505.
22. Danion JM, Rein W, Fleurot O, Group AS (1999): Improvement of schizophrenic patients with primary negative symptoms treated with amisulpride. *American Journal of Psychiatry*, pp 610-616.
23. Davidson M, Emsley R, Kramer M, Ford L, Pan G, Lim P, et al. (2007): Efficacy, safety and early response of paliperidone extended-release tablets (paliperidone ER): results of a 6-week, randomized, placebo-controlled study. *Schizophr Res*. 93:117-130.
24. Downing AM, Kinon BJ, Millen BA, Zhang L, Liu L, Morozova MA, et al. (2014): A double-blind, placebo-controlled comparator study of LY2140023 monohydrate in patients with schizophrenia. *BMC psychiatry*. 14:351.
25. Durgam S, Starace A, Li D, Migliore R, Ruth A, Nemeth G, et al. (2014): An evaluation of the safety and efficacy of cariprazine in patients with acute exacerbation of schizophrenia: a phase II, randomized clinical trial. *Schizophr Res*. 152:450-457.
26. Durgam S, Cutler AJ, Lu K, Migliore R, Ruth A, Laszlovszky I, et al. (2015): Cariprazine in acute exacerbation of schizophrenia: a fixed-dose, phase 3, randomized, double-blind, placebo- and active-controlled trial. *J Clin Psychiatry*. 76:e1574-1582.
27. Durgam S, Litman RE, Papadakis K, Li D, Németh G, Laszlovszky I (2016): Cariprazine in the treatment of schizophrenia: a proof-of-concept trial. *International clinical psychopharmacology*. 31:61.

28. Egan MF, Zhao X, Smith A, Troyer MD, Uebele VN, Pidkorytov V, et al. (2013): Randomized controlled study of the T-type calcium channel antagonist MK-8998 for the treatment of acute psychosis in patients with schizophrenia. *Human Psychopharmacology: Clinical and Experimental*. 28:124-133.
29. Fabre Jr LF, Arvanitis L, Pultz J, Jones VM, Malick JB, Slotnick VB (1995): ICI 204,636, a novel, atypical antipsychotic: early indication of safety and efficacy in patients with chronic and subchronic schizophrenia. *Clinical therapeutics*. 17:366-378.
30. Garcia E, Robert M, Peris F, Nakamura H, Sato N, Terazawa Y (2009): The efficacy and safety of blonanserin compared with haloperidol in acute-phase schizophrenia. *CNS drugs*. 23:615-625.
31. Goff DC, Posever T, Herz L, Simmons J, Kletti N, Lapierre K, et al. (1998): An exploratory haloperidol-controlled dose-finding study of ziprasidone in hospitalized patients with schizophrenia or schizoaffective disorder. *JClinPsychopharmacol*. 18:296-304.
32. Goff DC, McEvoy JP, Citrome L, Mech AW, Bustillo JR, Gil R, et al. (2013): High-dose oral ziprasidone versus conventional dosing in schizophrenia patients with residual symptoms: the ZEBRAS study. *J Clin Psychopharmacol*. 33:485-490.
33. Gopal S, Hough DW, Xu H, Lull JM, Gassmann-Mayer C, Remmerie BM, et al. (2010): Efficacy and safety of paliperidone palmitate in adult patients with acutely symptomatic schizophrenia: a randomized, double-blind, placebo-controlled, dose-response study. *Int Clin Psychopharmacol*. 25:247-256.
34. Hale A, Azorin JM, Kasper S, Maier W, Syvalahti E, van der Burght M, et al. (2000): Sertindole improves both the positive and negative symptoms of schizophrenia: Results of a phase III trial. *IntJPsychClinPract*. 4:55-62.
35. Heinrich K, Klieser E, Lehmann E, Ḱinzler E, Hruschka H (1994): Risperidone versus clozapine in the treatment of schizophrenic patients with acute symptoms: a double blind, randomized trial. *Progress in Neuro-Psychopharmacology and Biological Psychiatry*. 18:129-137.
36. 041-021 SH (2009): A multicenter, randomized, double-blind, fixed-dose, 6-week trial of the efficacy and safety of asenapine compared with placebo using olanzapine postive control in subjects with an acute exacerbation of schizophrenia.
37. Higuchi T, Ishigooka J, Iyo M, Yeh CB, Ebenezer EG, Liang KY, et al. (2019): Lurasidone in the treatment of schizophrenia: Results of a double-blind, placebo-controlled trial in Asian patients. *Asia-Pacific Psychiatry*. 11:e12352.
38. Higuchi T, Iyo M, Kwon JS, Chou YH, Chen HK, Chen JY, et al. (2019): Randomized, double-blind, placebo, and risperidone-controlled study of lurasidone in the treatment of schizophrenia: Results of an inconclusive 6-week trial. *Asia-Pacific Psychiatry*. 11:e12354.
39. Hirayasu Y, Tomioka M, Iizumi M, Kikuchi H (2010): A double-blind, placebo-controlled, comparative study of paliperidone extended release (ER) tablets in patients with schizophrenia. *Jpn J Clin Psychopharmacol*. 13:2077-2103.
40. Honer WG, MacEwan GW, Gendron A, Stip E, Labelle A, Williams R, et al. (2012): A randomized, double-blind, placebo-controlled study of the safety and tolerability of high-dose quetiapine in patients with persistent symptoms of schizophrenia or schizoaffective disorder. *J Clin Psychiatry*. 73:13-20.
41. Ishigooka J, Iwashita S, Tadori Y (2018): Efficacy and safety of brexpiprazole for the treatment of acute schizophrenia in Japan: A 6-week, randomized, double-blind, placebo-controlled study. *Psychiatry Clin Neurosci*. 72:692-700.
42. Iyo M, Ishigooka J, Nakamura M, Sakaguchi R, Okamoto K, Mao Y, et al. (2021): Efficacy and safety of lurasidone in acutely psychotic patients with schizophrenia: A 6-week, randomized, double-blind, placebo-controlled study. *Psychiatry Clin Neurosci*.

43. Kahn RS, Schulz SC, Palazov VD, Reyes EB, Brecher M, Svensson O, et al. (2007): Efficacy and tolerability of once-daily extended release quetiapine fumarate in acute schizophrenia: a randomized, double-blind, placebo-controlled study. *JClinPsychiatry*. 68:832-842.
44. Kane JM, Carson WH, Saha AR, McQuade RD, Ingenito GG, Zimbroff DL, et al. (2002): Efficacy and safety of aripiprazole and haloperidol versus placebo in patients with schizophrenia and schizoaffective disorder. *JClinPsychiatry*. 63:763-771.
45. Kane JM, Eerdekens M, Lindenmayer JP, Keith SJ, Lesem M, Karcher K (2003): Long-acting injectable risperidone: efficacy and safety of the first long-acting atypical antipsychotic. *Am J Psychiatry*. 160:1125-1132.
46. Kane JCFKMFLG-MCLPEM (2007): Treatment of schizophrenia with paliperidone extended-release tablets: a 6-week placebo-controlled trial. *Schizophrenia Research*. Netherlands, pp 147-161.
47. Kane JM, Cohen M, Zhao J, Alphs L, Panagides J (2010): Efficacy and safety of asenapine in a placebo- and haloperidol-controlled trial in patients with acute exacerbation of schizophrenia. *JClinPsychopharmacol*. 30:106-115.
48. Kane JM, Peters-Strickland T, Baker RA, Hertel P, Eramo A, Jin N, et al. (2014): Aripiprazole once-monthly in the acute treatment of schizophrenia: findings from a 12-week, randomized, double-blind, placebo-controlled study. *The Journal of clinical psychiatry*. 75:1254-1260.
49. Kane JM, Skuban A, Ouyang J, Hobart M, Pfister S, McQuade RD, et al. (2015): A multicenter, randomized, double-blind, controlled phase 3 trial of fixed-dose brexpiprazole for the treatment of adults with acute schizophrenia. *Schizophr Res*. 164:127-135.
50. Kane JM, Zukin S, Wang Y, Lu K, Ruth A, Nagy K, et al. (2015): Efficacy and Safety of Cariprazine in Acute Exacerbation of Schizophrenia: Results From an International, Phase III Clinical Trial. *J Clin Psychopharmacol*. 35:367-373.
51. Keck P, Buffenstein A, Ferguson J, Feighner J, Jaffe W, Harrigan EP, et al. (1998): Ziprasidone 40 and 120 mg/day in the acute exacerbation of schizophrenia and schizoaffective disorder: a 4-week placebo controlled trial. *Psychopharmacology*. 140:173-184.
52. King DJ, Link CG, Kowalcyk B (1998): A comparison of bd and tid dose regimens of quetiapine (seroquel) in the treatment of schizophrenia. *Psychopharmacology*, pp 139-146.
53. Kinon BJ, Volavka J, Stauffer V, Edwards SE, Liu-Seifert H, Chen L, et al. (2008): Standard and higher dose of olanzapine in patients with schizophrenia or schizoaffective disorder: a randomized, double-blind, fixed-dose study. *J Clin Psychopharmacol*. 28:392-400.
54. Kinon BJ, Zhang L, Millen BA, Osuntokun OO, Williams JE, Kollack-Walker S, et al. (2011): A multicenter, inpatient, phase 2, double-blind, placebo-controlled dose-ranging study of LY2140023 monohydrate in patients with DSM-IV schizophrenia. *Journal of clinical psychopharmacology*. 31:349-355.
55. Kinoshita T, Bai Y-M, Kim J-H, Miyake M, Oshima N (2016): Efficacy and safety of asenapine in Asian patients with an acute exacerbation of schizophrenia: a multicentre, randomized, double-blind, 6-week, placebo-controlled study. *Psychopharmacology*. 233:2663-2674.
56. Kramer M, Litman R, Hough D, Lane R, Lim P, Liu Y, et al. (2010): Paliperidone palmitate, a potential long-acting treatment for patients with schizophrenia. Results of a randomized, double-blind, placebo-controlled efficacy and safety study. *The international journal of neuropsychopharmacology*. 13:635-647.
57. Landbloom R, Mackle M, Wu X, Kelly L, Snow-Adami L, McIntyre RS, et al. (2017): Asenapine for the treatment of adults with an acute exacerbation of schizophrenia: results from a randomized, double-blind, fixed-dose, placebo-controlled trial with olanzapine as an active control. *CNS spectrums*. 22:333-341.
58. Lane H-Y, Chang W-H, Chiu C-C, Huang M-C, Lee S-H, Chen J-Y (2001): A pilot double-blind, dose-comparison study of risperidone in drug-naive, first-episode schizophrenia. *The Journal of clinical psychiatry*. 62:994.

59. Lauriello J, Lambert T, Andersen S, Lin D, Taylor CC, McDonnell D (2008): An 8-week, double-blind, randomized, placebo-controlled study of olanzapine long-acting injection in acutely ill patients with schizophrenia. *J Clin Psychiatry*. 69:790-799.
60. Lecrubier Y, Quintin P, Bouhassira M, Perrin E, Lancrenon S (2006): The treatment of negative symptoms and deficit states of chronic schizophrenia: olanzapine compared to amisulpride and placebo in a 6-month double-blind controlled clinical trial. *Acta Psychiatrica Scandinavica*. Denmark, pp 319-327.
61. Lieberman JA, Davis RE, Correll CU, Goff DC, Kane JM, Tamminga CA, et al. (2016): ITI-007 for the treatment of schizophrenia: a 4-week randomized, double-blind, controlled trial. *Biological psychiatry*. 79:952-961.
62. Lindenmayer JP, Brown D, Liu S, Brecher M, Meulien D (2008): The efficacy and tolerability of once-daily extended release quetiapine fumarate in hospitalized patients with acute schizophrenia: a 6-week randomized, double-blind, placebo-controlled study. *Psychopharmacol Bull*. 41:11-35.
63. Lindenmayer JP, Citrome L, Khan A, Kaushik S, Kaushik S (2011): A randomized, double-blind, parallel-group, fixed-dose, clinical trial of quetiapine at 600 versus 1200 mg/d for patients with treatment-resistant schizophrenia or schizoaffective disorder. *J Clin Psychopharmacol*. 31:160-168.
64. Litman RE, Smith MA, Doherty JJ, Cross A, Raines S, Gertsik L, et al. (2016): AZD8529, a positive allosteric modulator at the mGluR2 receptor, does not improve symptoms in schizophrenia: A proof of principle study. *Schizophrenia research*. 172:152-157.
65. Loebel A, Cucchiaro J, Sarma K, Xu L, Hsu C, Kalali AH, et al. (2013): Efficacy and safety of lurasidone 80 mg/day and 160 mg/day in the treatment of schizophrenia: a randomized, double-blind, placebo- and active-controlled trial. *Schizophr Res*. 145:101-109.
66. Loebel A, Silva R, Goldman R, Watabe K, Cucchiaro J, Citrome L, et al. (2016): Lurasidone Dose Escalation in Early Nonresponding Patients With Schizophrenia: A Randomized, Placebo-Controlled Study. *J Clin Psychiatry*.
67. Loo H, Poirier-Littre MF, Theron M, Rein W, Fleurot O (1997): Amisulpride versus placebo in the medium-term treatment of the negative symptoms of schizophrenia. *Br J Psychiatry*. 170:18-22.
68. Marder SR, Meibach RC (1994): Risperidone in the treatment of schizophrenia. *AmJPsychiatry*. 151:825-835.
69. Marder SR, Kramer M, Ford L, Eerdekens E, Lim P, Eerdekens M, et al. (2007): Efficacy and safety of paliperidone extended-release tablets: results of a 6-week, randomized, placebo-controlled study. *Biological Psychiatry*. 62:1363-1370.
70. McEvoy JP, Daniel DG, Carson WH, Jr., McQuade RD, Marcus RN (2007): A randomized, double-blind, placebo-controlled, study of the efficacy and safety of aripiprazole 10, 15 or 20 mg/day for the treatment of patients with acute exacerbations of schizophrenia. *JPsychiatrRes*. 41:895-905.
71. Meltzer HY, Arvanitis L, Bauer D, Rein W, Group M-TS (2004): Placebo-controlled evaluation of four novel compounds for the treatment of schizophrenia and schizoaffective disorder. *American Journal of Psychiatry*. 161:975-984.
72. Meltzer H, Barbato L, Heisterberg J, Yeung P, Shapira N (2007): A randomized, double-blind, placebo-controlled efficacy and safety study of bifeprunox as treatment for patients with acutely exacerbated schizophrenia. *Schizophrenia Bulletin*: OXFORD UNIV PRESS GREAT CLARENDON ST, OXFORD OX2 6DP, ENGLAND, pp 446-446.
73. Meltzer HY, Cucchiaro J, Silva R, Ogasa M, Phillips D, Xu J, et al. (2011): Lurasidone in the treatment of schizophrenia: a randomized, double-blind, placebo- and olanzapine-controlled study. *Am J Psychiatry*. 168:957-967.

74. Meltzer HY, Elkis H, Vanover K, Weiner DM, van Kammen DP, Peters P, et al. (2012): Pimavanserin, a selective serotonin (5-HT) 2A-inverse agonist, enhances the efficacy and safety of risperidone, 2 mg/day, but does not enhance efficacy of haloperidol, 2 mg/day: comparison with reference dose risperidone, 6 mg/day. *Schizophrenia research*. 141:144-152.
75. Meltzer H, Lindenmayer J-P, Kwentus J, Share D, Johnson R, Jayathilake K (2014): A six month randomized controlled trial of long acting injectable risperidone 50 and 100 mg in treatment resistant schizophrenia. *Schizophrenia research*. 154:14-22.
76. Meltzer HY, Share DB, Jayathilake K, Salomon RM, Lee MA (2020): Lurasidone Improves Psychopathology and Cognition in Treatment-Resistant Schizophrenia. *Journal of clinical psychopharmacology*. 40:240.
77. Merlo M, Hofer H, Gekle W, Berger G, Ventura J, Panhuber I, et al. (2002): Risperidone, 2 mg/day vs. 4 mg/day, in first-episode, acutely psychotic patients: treatment efficacy and effects on fine motor functioning. *The Journal of clinical psychiatry*. 63:885-891.
78. Nakamura M, Ogasa M, Guarino J, Phillips D, Severs J, Cucchiaro J, et al. (2009): Lurasidone in the treatment of acute schizophrenia: a double-blind, placebo-controlled trial. *J Clin Psychiatry*. 70:829-836.
79. Nakamura T, Kubota T, Iwakaji A, Imada M, Kapás M, Morio Y (2016): Clinical pharmacology study of cariprazine (MP-214) in patients with schizophrenia (12-week treatment). *Drug design, development and therapy*. 10:327.
80. Nasrallah HA, Gopal S, Gassmann-Mayer C, Quiroz JA, Lim P, Eerdekens M, et al. (2010): A controlled, evidence-based trial of paliperidone palmitate, a long-acting injectable antipsychotic, in schizophrenia. *Neuropsychopharmacology*. 35:2072-2082.
81. Nasrallah HA, Silva R, Phillips D, Cucchiaro J, Hsu J, Xu J, et al. (2013): Lurasidone for the treatment of acutely psychotic patients with schizophrenia: a 6-week, randomized, placebo-controlled study. *J Psychiatr Res*. 47:670-677.
82. Nasser AF, Henderson DC, Fava M, Fudala PJ, Twumasi-Ankrah P, Kouassi A, et al. (2016): Efficacy, safety, and tolerability of RBP-7000 once-monthly risperidone for the treatment of acute schizophrenia: an 8-week, randomized, double-blind, placebo-controlled, multicenter phase 3 study. *Journal of clinical psychopharmacology*. 36:130-140.
83. NCT00563706 Study Evaluating Vabicaserin in Subjects With Schizophrenia. https://ClinicalTrials.gov/show/NCT00563706.
84. Correll CU, Skuban A, Hobart M, Ouyang J, Weiller E, Weiss C, et al. (2016): Efficacy of brexpiprazole in patients with acute schizophrenia: Review of three randomized, double-blind, placebo-controlled studies. *Schizophr Res*. 174:82-92.
85. NCT01625000 Safety and Efficacy of MP-214 in Patients With Schizophrenia. https://ClinicalTrials.gov/show/NCT01625000.
86. NCT02469155 A Trial to Assess the Antipsychotic Efficacy of ITI-007 Over 6 Weeks of Treatment. https://ClinicalTrials.gov/show/NCT02469155.
87. Ogasa M, Kimura T, Nakamura M, Guarino J (2013): Lurasidone in the treatment of schizophrenia: a 6-week, placebo-controlled study. *Psychopharmacology (Berl)*. 225:519-530.
88. Oosthuizen P, Emsley R, Jadri Turner H, Keyter N (2004): A randomized, controlled comparison of the efficacy and tolerability of low and high doses of haloperidol in the treatment of first-episode psychosis. *International Journal of Neuropsychopharmacology*. 7:125-131.
89. Pandina GJ, Lindenmayer JP, Lull J, Lim P, Gopal S, Herben V, et al. (2010): A randomized, placebo-controlled study to assess the efficacy and safety of 3 doses of paliperidone palmitate in adults with acutely exacerbated schizophrenia. *J Clin Psychopharmacol*. 30:235-244.
90. Patil ST, Zhang L, Martenyi F, Lowe SL, Jackson KA, Andreev BV, et al. (2007): Activation of mGlu2/3 receptors as a new approach to treat schizophrenia: a randomized Phase 2 clinical trial. *Nature medicine*. 13:1102-1107.

91. Peuskens J, Group. RS (1995): Risperidone in the treatment of patients with chronic schizophrenia: a multi-national, multi-centre, double-blind, parallel-group study versus haloperidol. *BrJPsychiatry*. 166:712-726.
92. Potkin SG, Saha AR, Kujawa MJ, Carson WH, Ali M, Stock E, et al. (2003): Aripiprazole, an antipsychotic with a novel mechanism of action, and risperidone vs placebo in patients with schizophrenia and schizoaffective disorder. *ArchGenPsychiatry*. 60:681-690.
93. Potkin SG, Cohen M, Panagides J (2007): Efficacy and tolerability of asenapine in acute schizophrenia: a placebo-and risperidone-controlled trial. *The Journal of clinical psychiatry*. 68:1492-1500.
94. Potkin SG, Litman RE, Torres R, Wolfgang CD (2008): Efficacy of iloperidone in the treatment of schizophrenia: initial phase 3 studies. *JClinPsychopharmacol*. 28:S4-11.
95. 049 S (2010): A 6-week, double-blind, randomized, fixed dose, parallel-group study of the efficacy and safety of three dose levels of SM-13496 (lurasidone) compared to placebo and haloperidol in patients with schizophrenia who are experiencing an acute exacerbation of symptoms.
96. Puech A, Fleurot O, Rein W (1998): Amisulpride, an atypical antipsychotic, in the treatment of acute episodes of schizophrenia: a dose-ranging study vs. haloperidol. *Acta PsychiatrScand*. 98:65-72.
97. Santos JL, Cabranes JA, Vazquez C, Fuentenebro F, Almoguera I, Ramos JA (1989): Clinical response and plasma haloperidol levels in chronic and subchronic schizophrenia. *Biological psychiatry*. 26:381-388.
98. Schmidt ME, Kent JM, Daly E, Janssens L, Van Osselaer N, Hüsken G, et al. (2012): A double-blind, randomized, placebo-controlled study with JNJ-37822681, a novel, highly selective, fast dissociating D2 receptor antagonist in the treatment of acute exacerbation of schizophrenia. *European Neuropsychopharmacology*. 22:721-733.
99. Simpson G, Angus J, Edwards J (1967): A controlled study of haloperidol in chronic schizophrenia. *Current therapeutic research, clinical and experimental*. 9:407.
100. 104 S (1998): Center for drug evaluation and research approval package for application number 20-825. Medical review. *http://wwwfdagov*.
101. 115 2000 S (2000): Center for drug evaluation and research approval package for application number 20-825. Medical review. http://www.fda.gov.
102. 128-301 S (1997): Study report of study 128-301. Pfizer, data on file.
103. 2002 S9 (2002): Center for drug evaluation and research. Application number 21-436. Medical review(s). http://www.fda.gov.
104. 1996 SR-U-7 (1996): Office of Clinical pharmacology and biopharmacy review. NDA number: 20272. *Janssen-Cilag, data on file*.
105. Takahashi N, Takahashi M, Saito T, Iizumi M, Saito Y, Shimizu H, et al. (2013): Randomized, placebo-controlled, double-blind study assessing the efficacy and safety of paliperidone palmitate in Asian patients with schizophrenia. *Neuropsychiatric disease and treatment*. 9:1889.
106. Van Kammen DP, McEvoy JP, Targum SD, Kardatzke D, Sebree TB (1996): A randomized, controlled, dose-ranging trial of sertindole in patients with schizophrenia. *Psychopharmacology*. 124:168-175.
107. DeMartinis N, Banerjee A, Kumar V, Boyer S, Schmidt C, Arroyo S (2012): Poster# 212 results of a phase 2a proof-of-concept trial with a PDE10A Inhibitor in the treatment of acute exacerbation of schizophrenia. *Schizophrenia Research*.S262.
108. Zborowski J, Schmitz P, Staser J, O'Neil J, Giles K, Wallin B, et al. (1995): Efficacy and safety of sertindole in a trial of schizophrenic patients. *BiolPsychiatry*. 37:661-662.

109. Zimbroff DL, Kane JM, Tamminga CA, Daniel DG, Mack RJ, Wozniak PJ, et al. (1997): Controlled, dose response study of sertindole and haloperidol in the treatment of schizophrenia. *AmJPsychiatry*. 154:782-791.

eAppendix-5: Risk of bias assessments

The overall risk of bias was according to Furukawa et al 2016 (1).

Table of risk of bias assessments for individual studies (Cochrane risk of bias tool 1)

Study	Random sequence generation	Allocation concealment	Blinding of participants and personnel	Blinding of outcome assessment	Incomplete outcome data	Selective reporting	Other bias	Overall
Arvanitis 1997	Unclear	Unclear	Unclear	Unclear	Low	Low	Low	Moderate
Barnas 2001	Unclear	Low	High	Low	Unclear	High	Low	High
Beasley 1996a	Low	Low	Low	Low	Low	Low	Low	Low
Beasley 1996b	Low	Low	Unclear	Unclear	Low	Low	Low	Low
Beasley 1997	Low	Low	Low	Low	Low	Low	Low	Low
Boyer 1995	Low	Low	Unclear	Unclear	High	High	Low	High
Cantillon 2014	Unclear	Unclear	Unclear	Unclear	Unclear	Unclear	Low	Moderate
Canuso 2010b	Low	Low	Low	Low	Low	Low	Low	Low
Casey 2008	Unclear	Unclear	Unclear	Unclear	Low	High	Low	Moderate
Chouinard 1993	Unclear	Unclear	Low	Low	Low	Low	Low	Low
Citrome 2021	Low	Low	Low	Low	Low	High	Low	Moderate
Cooper 2000a	Unclear	Unclear	Low	Low	Low	Low	Low	Low
Coppola 2011	Low	Low	Low	Low	Low	Low	Low	Low
Correll 2015	Low	Unclear	Unclear	Unclear	Low	Low	Low	Low
Correll 2020	Low	Low	Low	Low	Low	Low	Low	Low
Correll 2020b	Low	Low	Low	Low	Low	Low	Low	Low
Corrigan 2004	Low	Low	Low	Low	Low	High	Low	Moderate
Cutler 2006	Unclear	Unclear	Low	Low	Low	Low	Low	Low
Cutler 2008	Low	Low	Low	Low	High	Low	Low	Moderate
Cutler 2008a	Unclear	Unclear	Low	Low	Low	Low	Low	Low
Daniel 1999	Low	Unclear	Unclear	Unclear	Low	Low	Low	Low

Danion 1999	Unclear	Unclear	Unclear	Unclear	Low	Low	Low	Moderate
Davidson 2007	Low	Low	Low	Low	Low	Low	Low	Low
Downing 2014	Unclear	Unclear	Low	Low	Low	Low	Low	Low
Durgam 2014	Unclear	Unclear	Unclear	Unclear	Low	Low	Low	Moderate
Durgam 2015	Unclear	Unclear	Unclear	Unclear	Low	Low	Low	Moderate
Durgam 2016a	Unclear	Unclear	Unclear	Unclear	Low	Unclear	Low	Moderate
Egan 2013	Low	Low	Low	Low	Low	Low	Low	Low
Fabre 1995	Unclear	Unclear	Unclear	Unclear	Low	Low	Low	Moderate
Garcia 2009	Low	Unclear	Low	Low	Low	High	Low	Moderate
Goff 1998	Unclear	Unclear	Unclear	Unclear	Low	Low	Low	Moderate
Goff 2013	Unclear	Unclear	Low	Low	Unclear	Low	Low	Low
Gopal 2010	Low	Low	Low	Low	Low	Low	Low	Low
Hale 2000	Low	Low	Low	Low	Low	Low	Low	Low
Heinrich 1994	Unclear	Unclear	Unclear	Unclear	Low	Low	Low	Moderate
Hera 041-021	Unclear	Unclear	Low	Unclear	Unclear	Unclear	Unclear	Moderate
Higuchi 2019a	Low	Unclear	Low	Low	Low	Low	Low	Low
Higuchi 2019b	Unclear	Unclear	Unclear	Unclear	Low	Low	Low	Moderate
Hirayasu 2010	Unclear	Unclear	Low	Low	Unclear	Unclear	Low	Moderate
Honer 2010	Low	Low	Unclear	Unclear	Low	Low	Low	Low
Ishigooka 2018	Low	Low	Unclear	Unclear	Low	Low	Low	Low
Iyo 2021	Low	Low	Low	Low	Low	Low	Low	Low
Kahn 2007	Unclear	Unclear	Low	Low	Low	Low	Low	Low
Kane 2002	Unclear	Unclear	Unclear	Unclear	Low	Low	Low	Moderate
Kane 2003	Low	Low	Low	Low	Low	Low	Low	Low
Kane 2007b	Low	Low	Low	Low	Low	Low	Low	Low
Kane 2010a	Unclear	Unclear	Low	Low	Unclear	Low	Low	Low
Kane 2014	Low	Low	Low	Low	Low	Low	Low	Low
Kane 2015a	Low	Low	Low	Low	Low	Low	Low	Low
Kane 2015b	Unclear	Unclear	Unclear	Unclear	Low	Low	Low	Moderate

Keck 1998	Low	Unclear	Unclear	Unclear	Low	Low	Low	Low
King 1998	Low	Low	Unclear	Unclear	Low	Low	Low	Low
Kinon 2006c	Low	Low	Low	Low	Low	Low	Low	Low
Kinon 2011	Low	Low	Low	Low	High	Low	Low	Moderate
Kinoshita 2016	Low	Low	Low	Low	Low	Low	Low	Low
Kramer 2010	Low	Low	High	High	Low	Low	Low	High
Landbloom 2016	Low	Unclear	Low	Low	Low	Low	Low	Low
Lane 2001	Unclear	Unclear	Unclear	Unclear	Low	Unclear	Unclear	Moderate
Lauriello 2008	Low	Low	Unclear	Unclear	Low	Low	Low	Low
Lecrubier 2006	Low	Low	Low	Low	High	High	Low	High
Liebermann 2015	Low	Low	Low	Low	Low	Low	Low	Low
Lindenmayer 2008	Low	Low	Low	Low	Low	Low	Low	Low
Lindenmayer 2011	Unclear	Unclear	Low	Low	Low	High	Low	Moderate
Litman 2016	Low	Unclear	Low	Low	Low	Low	Low	Low
Loebel 2013	Low	Low	Low	Low	Low	Low	Low	Low
Loebel 2015a	Low	Low	Low	Low	Low	Low	Low	Low
Loo 1997	Low	Low	Low	Low	Low	High	High	High
Marder 1994	Low	Unclear	Low	Low	Low	Low	Low	Low
Marder 2007c	Low	Low	Low	Low	Low	Low	Low	Low
McEvoy 2007b	Low	Low	Low	Low	Low	Low	Low	Low
Meltzer 2004	Low	Unclear	Unclear	Unclear	Low	Low	Low	Low
Meltzer 2007a	Unclear	Unclear	Unclear	Unclear	Low	Unclear	Unclear	Moderate
Meltzer 2011	Low	Low	Low	Low	Low	Low	Low	Low
Meltzer 2012	Unclear	Unclear	Unclear	Unclear	Low	Low	Low	Moderate
Meltzer 2014	Unclear	Unclear	Unclear	Unclear	Unclear	Unclear	Low	Moderate
Meltzer 2020	Unclear	Unclear	Low	Low	Low	Low	Low	Low
Merlo 2000	Unclear	Unclear	Unclear	Unclear	Unclear	Unclear	Unclear	Moderate
Nakamura 2009	Low	Low	Low	Low	Low	Low	Low	Low
Nakamura 2016	Unclear	Unclear	High	High	Low	Low	Low	High

Nasrallah 2010	Low	Low	Low	Low	Low	Low	Low	Low
Nasrallah 2013	Low	Low	Low	Low	Low	Low	Low	Low
Nasser 2016	Low	Low	Low	Low	Low	Low	Low	Low
NCT00563706	Unclear	Unclear	Low	Low	Low	Low	Low	Low
NCT00905307	Low	Low	Low	Low	Low	Low	Low	Low
NCT01625000	Unclear	Unclear	Unclear	Unclear	High	Unclear	Low	Moderate
NCT02469155	Unclear	Unclear	Low	Low	Low	Unclear	Unclear	Moderate
Ogasa 2012	Low	Low	Low	Low	Low	Low	Low	Low
Oosthuizen 2004	Unclear	Unclear	Low	Low	Low	Low	Low	Low
Pandina 2010	Low	Low	Low	Low	Unclear	Unclear	Low	Low
Patil 2007	Unclear	Unclear	Unclear	Unclear	Low	High	Low	Moderate
Peuskens 1995	Low	Low	Low	Low	Low	Low	Low	Low
Potkin 2003	Unclear	Unclear	Unclear	Unclear	Low	High	Low	Moderate
Potkin 2007c	Unclear	Unclear	Low	Low	Low	Low	Low	Low
Potkin 2008a	Low	Low	Low	Low	Low	Low	Low	Low
Potkin 2015	Low	Low	Low	Low	Low	Low	Low	Low
Puech 1998	Low	Low	Low	Low	Low	Low	Low	Low
Santos 1989	Unclear	Unclear	High	High	High	High	Low	High
Schmidt 2014	Low	Low	Low	Low	Low	High	Low	Moderate
Simpson 1967	Unclear	Unclear	Unclear	Unclear	Unclear	High	Low	Moderate
Study 104	Unclear	Unclear	Unclear	Unclear	Low	Unclear	Unclear	Moderate
Study 115 2000	Low	Unclear	Unclear	Unclear	Low	Unclear	Low	Moderate
Study 128-301 1997	Unclear	Unclear	Unclear	Unclear	Unclear	Unclear	Unclear	Moderate
Study 93202 2002	Unclear	Unclear	Unclear	Unclear	Low	Low	Low	Moderate
Study 94202 2002	Unclear	Unclear	Low	Low	Low	Low	Low	Low
Study RIS-USA-72 1996	Unclear	Unclear	Low	Low	Low	Low	Low	Low
Takahashi 2013	Unclear	Unclear	Low	Low	Low	Low	Low	Low
Van Kammen 1996	Low	Unclear	Unclear	Unclear	Low	Low	Low	Low

Walling 2019	Low	Low	Low	Low	Low	Low	Low	Low
Zborowski 1995	Low	Low	Low	Low	Low	Low	Low	Low
Zimbroff 1997	Low	Low	Unclear	Unclear	Low	High	Low	Moderate

Summary of risk of bias of included studies assessed by Cochrane risk of bias tool 1

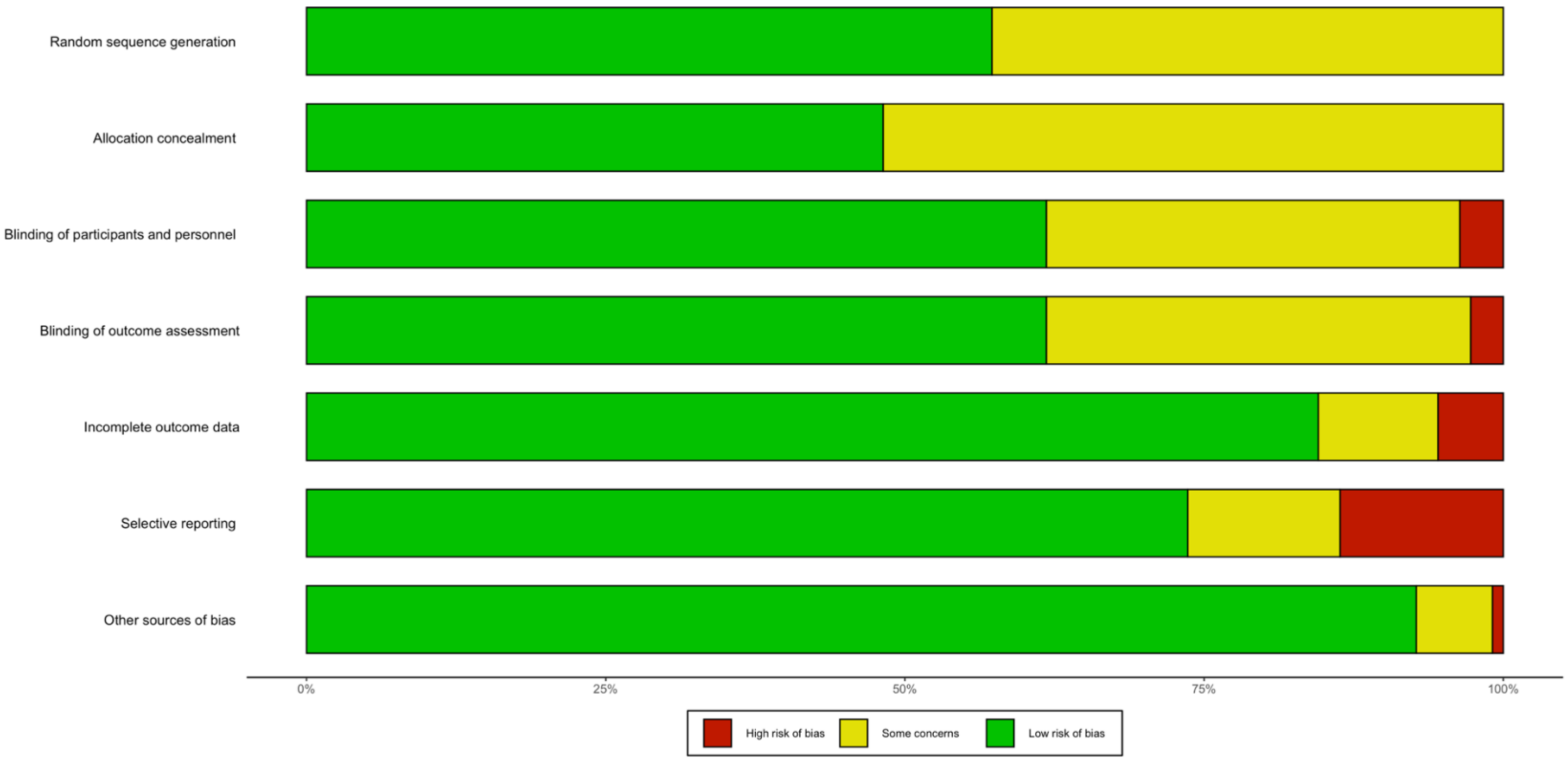

References

1. Furukawa TA, Salanti G, Atkinson LZ, Leucht S, Ruhe HG, Turner EH, et al. (2016): Comparative efficacy and acceptability of first-generation and second-generation antidepressants in the acute treatment of major depression: protocol for a network meta-analysis. *BMJ open*. 6:e010919.

eAppendix-6: Heterogeneity assessments

Heterogeneity was quantified at different dose levels using the variance partition coefficient (VPC) (1). We reported the median VPC and the corresponding interquartile range (IQR). We considered substantial heterogeneity when the median VPC was over 50%.

We have data mainly for the subgroup of people with acute exacerbation of chronic schizophrenia. In this subgroup, there was substantial heterogeneity in the dose-response curves for aripiprazole (median VPC=52%), and to a lesser degree but with a median VPC>40% for haloperidol (48.2%), cariprazine (45.6%), zotepine (40.9%). There was only one study for amisulpride for this subgroup and heterogeneity could not be assessed.

For the subgroup of predominant negative symptoms, 4 amisulpride studies and 1 olanzapine study were included. Heterogeneity was assessed as low in amisulpride studies (median VPC=0%). There was only one study for olanzapine for this subgroup and heterogeneity could not be assessed.

For the subgroup of first-episode schizophrenia, 2 risperidone studies with 4 distinct dose arms were included. The median VPC was 19.4%.
Details are provided below.

Variance partition coefficients (VPC) for dose-response curves of individual antipsychotics

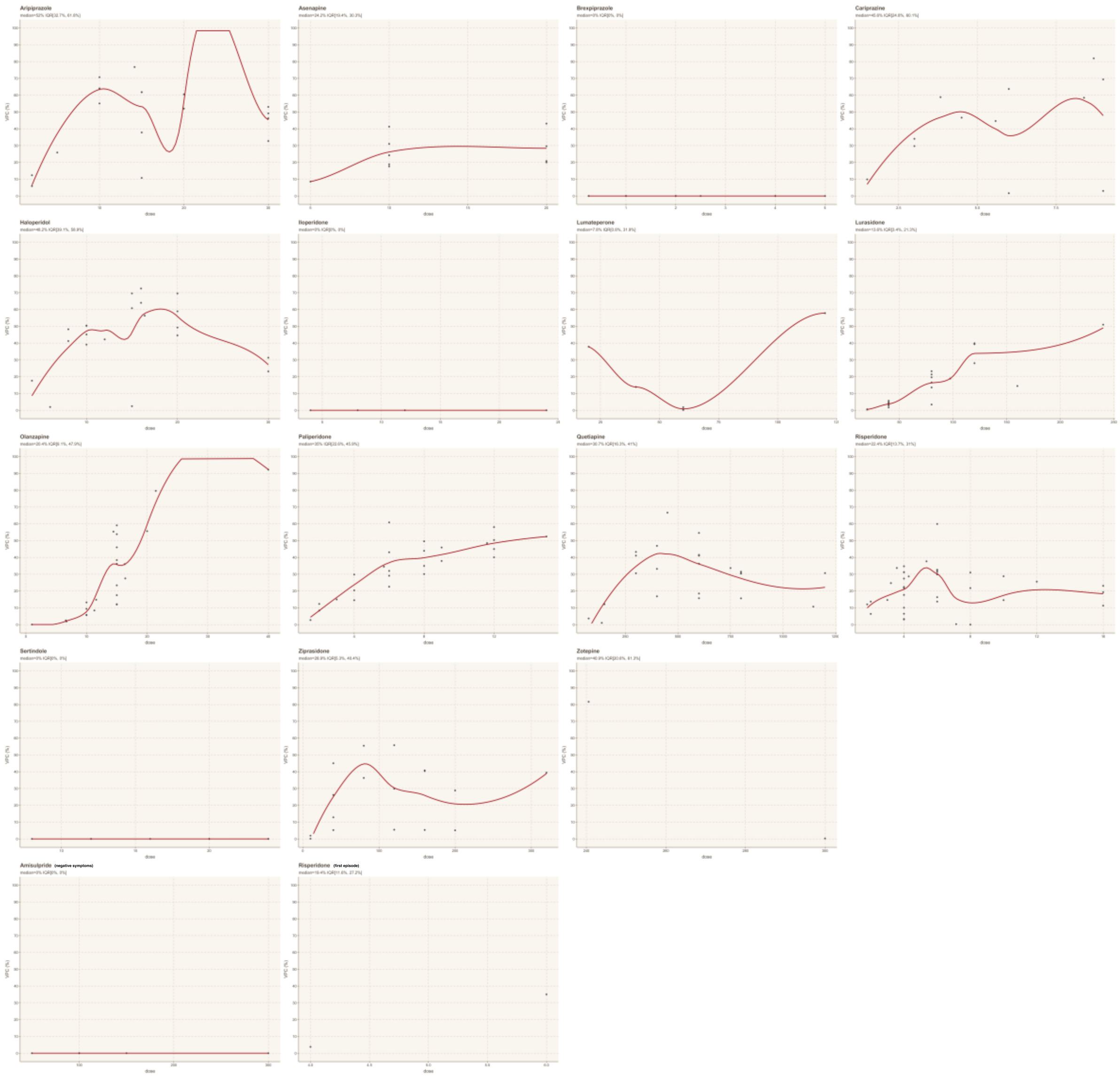

The red line represents a local polynomial regression to fit the VPC across doses. There were not enough data for zotepine and risperidone (first-episode) to draw a regression line. There was only one study for amisulpride (chronically-ill patients with acute exacerbation, not presented here), and the VPC could not be estimated.

Reference

1. Crippa A, Discacciati A, Bottai M, Spiegelman D, Orsini N (2019): One-stage dose–response meta-analysis for aggregated data. *Statistical methods in medical research*. 28:1579-1596.

eAppendix-7: Sensitivity analyses

Separate dose-response meta-analyses for continuous and dichotomous outcomes of extrapyramidal side-effects

We conducted separate analyses for the different outcome measures contributing to the main analysis:

1. Analysing number of participants used at least once antiparkinsonian medication (OR)
2. Analysing number of participants with at least one treatment emergent EPS (OR)
3. Analysing mean change from baseline to endpoint of validated rating scales (SMD) (to allow presentation in the same figure, SMDs were converted to ORs)

The findings are presented in the relevant panel plot below. The panel plot shows the dose-response curves for continuous and dichotomous outcomes of extrapyramidal side-effects. The black curve represents the main analysis as the reference. In the main analysis, we used data according to the following hierarchy: 1) number of participants with at least once antiparkinsonian medication, and when not available, 2) number of participants with at least one extrapyramidal side-effect, and when not available, 3) mean change of validated rating scales of extrapyramidal side-effects (converted to ORs, see “Methods and Materials” and eAppendix-2).
The red curve represents the analysis of number of participants used at least once antiparkinsonian medication (analysed with OR).
The purple curve represents number of participants with at least one treatment emergent EPS.
The blue represents mean change from baseline to endpoint of validated rating scales (analyzed with SMD but converted to OR in order to allow presentation in the same figure, see “Methods and Materials” and eAppendix-2).

The results did not materially change across different outcomes and most of them had similar shapes. We could expect some differences between outcomes, especially in those with low/very low confidence of evidence, such as amisulpride. Particularly, ORs derived from scale-derived data tended to be smaller than from number of patients receiving of antiparkinsonian medication or with at least one EPS. This could be potentially explained by the presence of skewness (e.g., large SDs that could dilute SMDs) and another possible explanation could be some EPS were transient, when using the mean change from baseline to endpoint as an estimate, the transient effect could be self-relieved and not recorded by the rating scales at the end of the study. However, self-reported or clinician-rated side-effect events were recorded when appeared, which could lead to higher estimation than endpoint data.

Different formulations of antipsychotics

We analyzed in the different formulations separately in a sensitivity analysis:

1. Asenapine oral and transdermal patch (HP3070)
2. Olanzapine oral and long-acting injectable (LAI)
3. Paliperidone oral and LAI

4. Quetiapine immediate (IR) and extended release (XR)
5. Risperidone oral and LAIs (consta, ISM, RBP-7000)

Note: only one study (Kane 2004) provided data for aripiprazole LAI, comparing a single dose of aripiprazole maintena with placebo, thus the dose response curve cannot be plotted. There were also no data for aripiprazole lauroxil.

The findings are presented in the relevant panel plot below. We had only one study on asenapine transdermal patch (HP3070), one on olanzapine LAI, two risperidone consta, one risperidone ISM, one risperidone RBP-7000. Although the comparisons with their oral formulations revealed some differences, we could not draw any conclusions due to sparse data.

Dose-response curves of paliperidone oral and LAI (once-monthly injection) generally agreed with each other, yet data at higher doses of LAI were not available.

The comparison between quetiapine IR and XR was restricted under 800mg/day due to lack of data. Quetiapine XR showed increased risk of EPS than IR with a bell-shaped curve. Quetiapine IR has a short half-life and XR could allow less frequent dosing due to its prolonged plasma concentration than IR. As we mentioned above, using the number of participants who used antiparkinsonian medication or with EPS could possibly record more transient events. It is plausible that transient events from XR would be more than IR because of prolonged plasma concentration. Nevertheless, future studies are needed to further investigate potential differences between quetiapine formulations.

Excluding studies with certain characteristics and using different knot points

We conducted the following sensitivity analyses:

1. Excluding non-dose-finding RCTs, i.e., studies comparing a single fixed-dose arm of antipsychotic drug to placebo
2. Excluding open RCTs
3. Excluding studies in people with treatment resistant schizophrenia
4. Setting knot points at 10th, 50th and 90th percentiles.

Note: Knot points at 10th, 50th and 90th percentiles were used for the analysis of asenapine, given that separate knots could not be defiend with 25th, 50th and 75th percentiles.

The findings are presented in the relevant panel plot below. The results were largely unchanged.

Separate dose-response meta-analyses for continuous and dichotomous outcomes of extrapyramidal side-effects

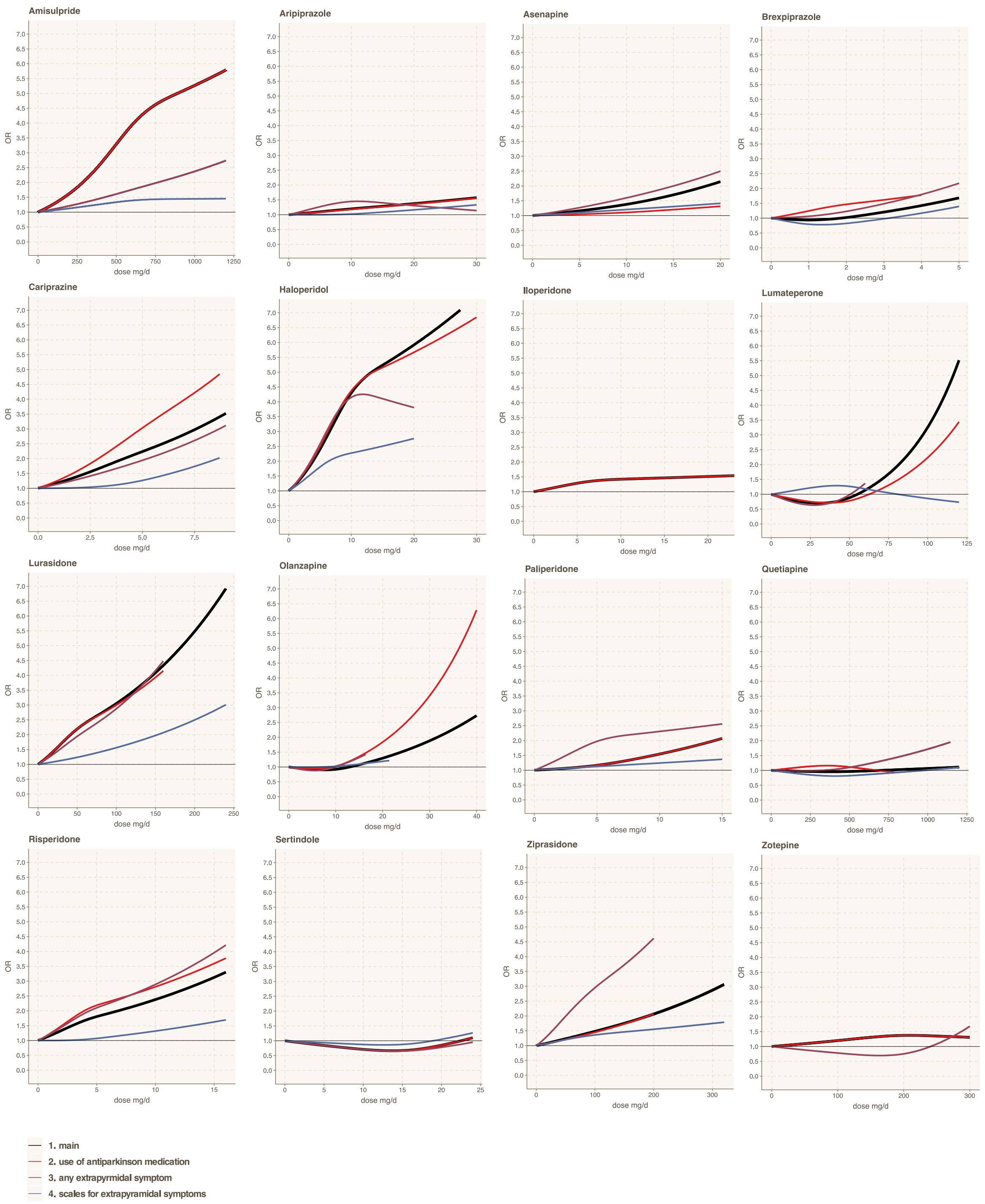

Dose-response curves of different formulations of antipsychotics

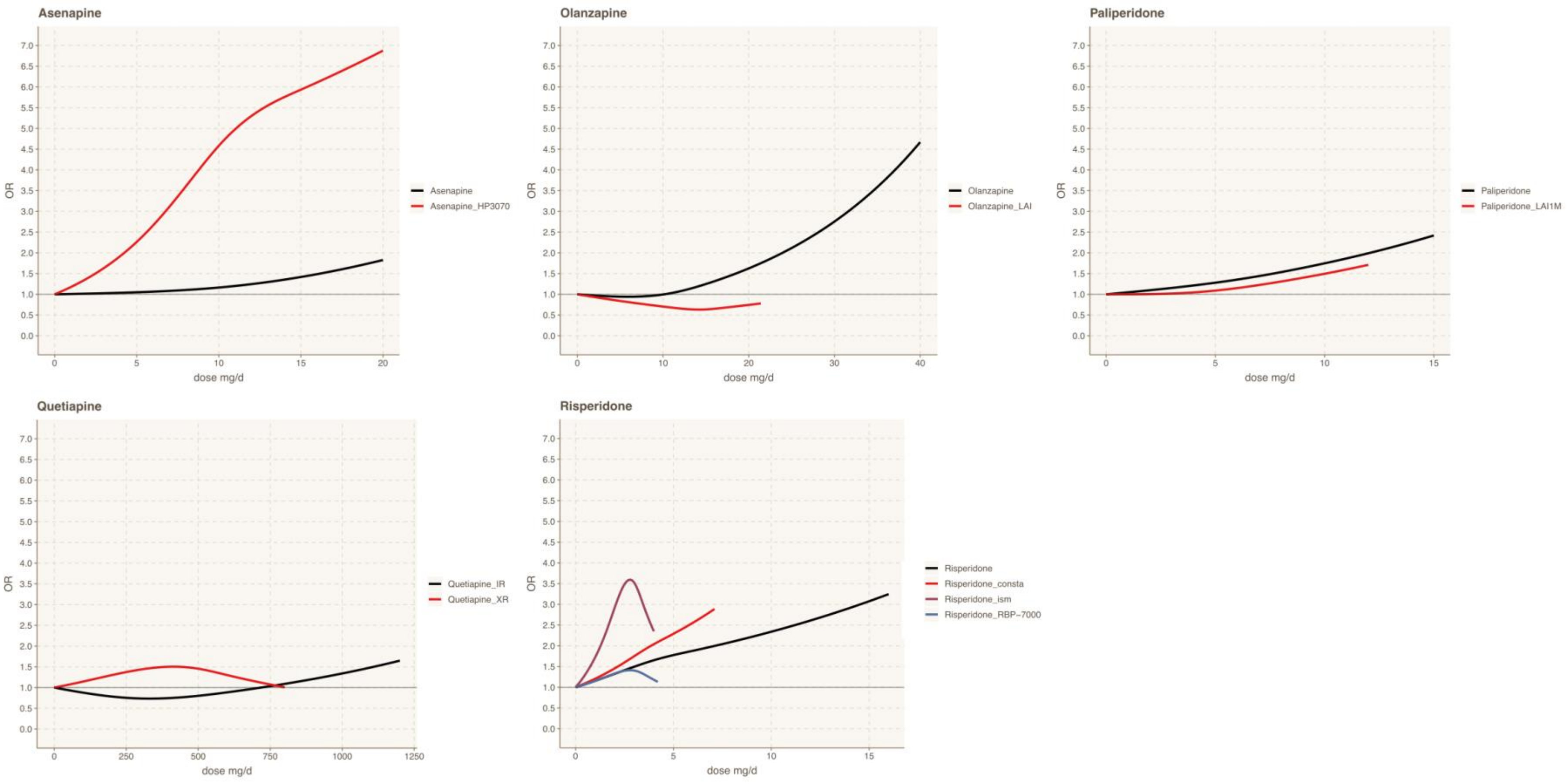

Dose-response curves of sensitivity analyses of excluding non-dose-finding RCTs, open RCTs, studies in treatment resistant schizophrenia, and of using different knot points

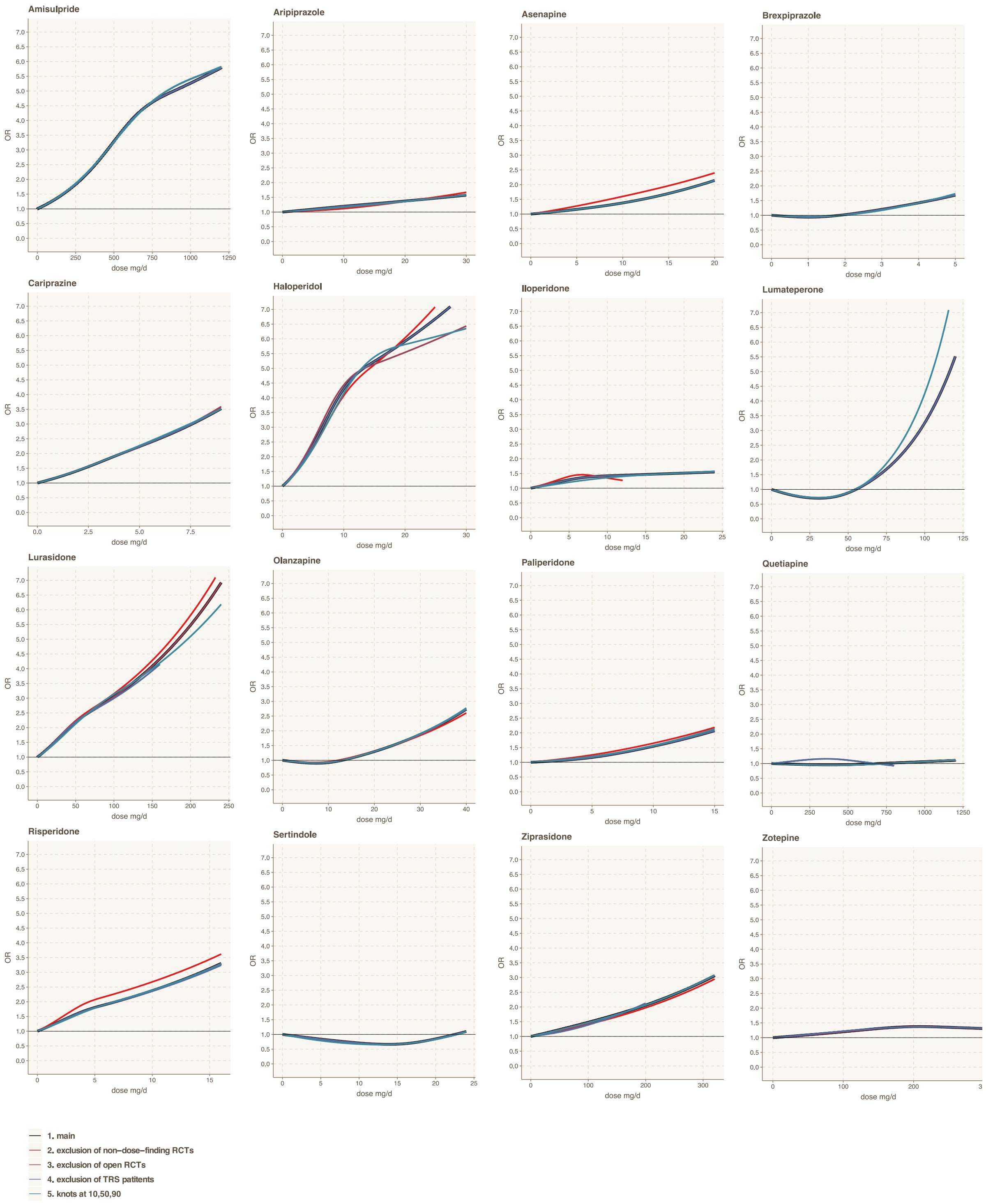

eAppendix-8: Small-study effects assessment

We assessed small-study effects, when there were at least 10 studies available,[1] namely haloperidol (15 studies), lurasidone (10), olanzapine (14), paliperidone (11) and risperidone (19).

First, we assessed the symmetry funnel plots of the pairwise comparisons of antipsychotics (any dose combined) versus placebo by visual inspection and with a meta-regression for sample size (sqrt transformed), given that there is correlation between ORs and their standard errors (1). Other methods, e.g., the Peter's test (1), could not be used, given that when dichotomous data were not available, they were estimated from the continuous, and thus, they did not report data on the number of events in the experimental and control groups. The forest plots, funnel plots and meta-regressions are presented below for each drug. There was indication of small-study effects for lurasidone (z-value=2.28, p-value=0.023).

Second, we conducted a dose-response meta-regression for study sample size (sqrt transformed) in a similar way to a previous dose-response meta-regression analysis (2). We assessed the impact of the sample size on the dose-response curve by conducting a Wald-type test for the two regression coefficients of the interactions between sample size and the two coefficients of the restricted cubic spline (3). Potential small-study effects were detected for lurasidone (χ^2=6.5, df=2, p=0.039).

More detailed findings of are presented below.

Haloperidol

Forest plot

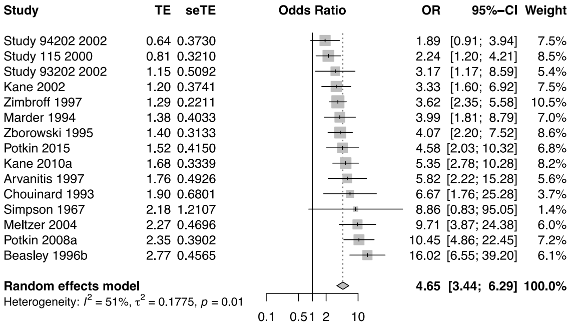

Funnel plot

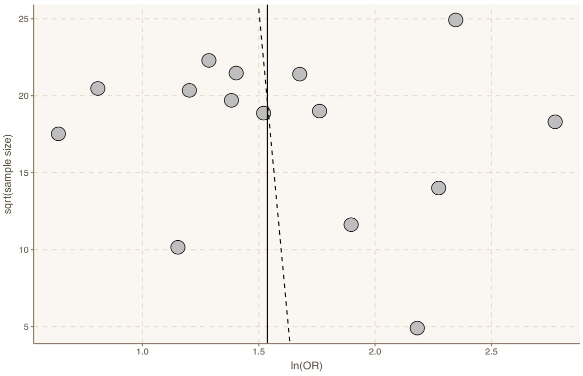

There were 15 placebo-controlled studies. No clear asymmetry was detected in the funnel plot and meta-regression (z-value=-0.155, p-value=0.877). The solid line in the funnel plot represents the pooled estimate from the meta-analysis, while the dotted line the meta-regression for sqrt(sample size).

No impact of the sample size on the dose-response curve was detected (χ^2=1.3, df=2, p=0.55).

Lurasidone

Forest plot

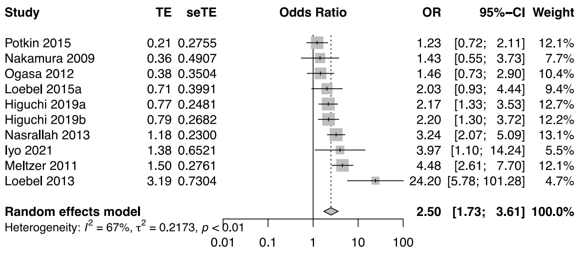

Funnel plot

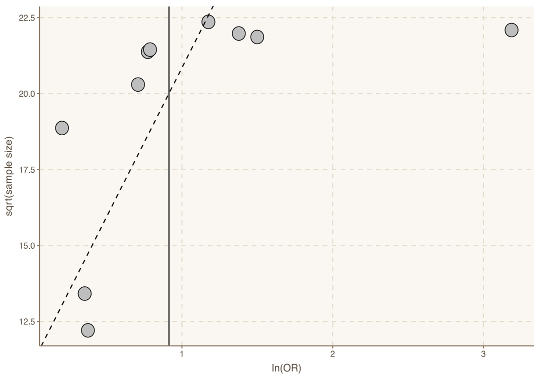

There were 10 placebo-controlled studies. The funnel plot indicated that smaller studies with higher ORs for extrapyramidal side-effects may be missing (z-value=0.102, p-value=0.023).
The solid line in the funnel plot represents the pooled estimate from the meta-analysis, while the dotted line the meta-regression for sqrt(sample size).

Sample size seemed to have an impact on the dose-response curve (χ^2=6.5, df=2, p=0.039).

Olanzapine

Forest plot

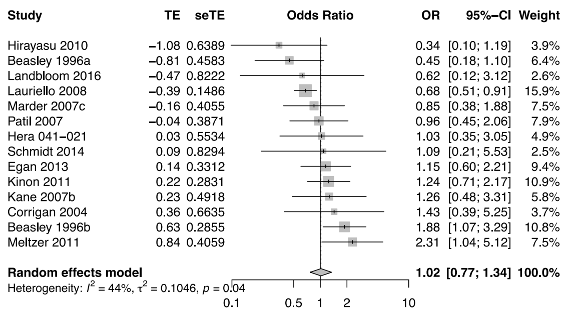

Funnel plot

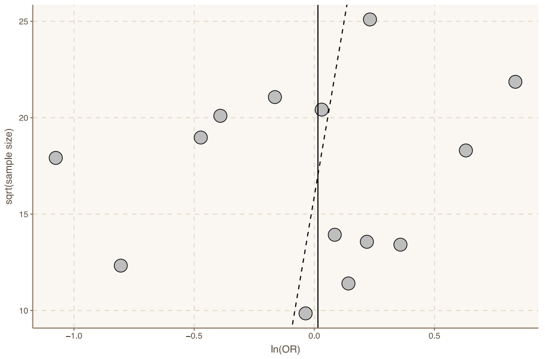

There were 14 placebo-controlled studies. No clear asymmetry was detected in the funnel plot and meta-regression (z-value=0.410, p-value = 0.682). The solid line in the funnel plot represents the pooled estimate from the meta-analysis, while the dotted line the meta-regression for sqrt(sample size).

No impact of the sample size on the dose-response curve was detected (χ^2=4.0, df=2, p=0.14).

Paliperidone

Forest plot

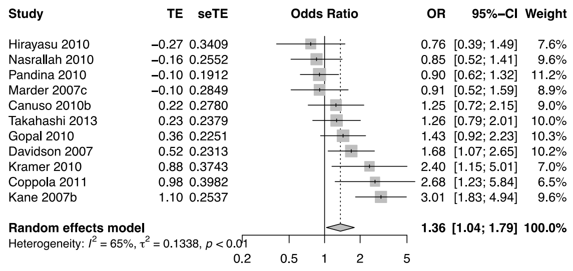

Funnel plot

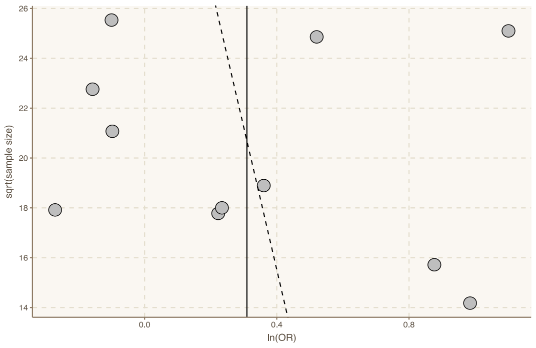

There were 11 placebo-controlled studies. No clear asymmetry was detected in the funnel plot and meta-regression (z-value=-0.443, p-value = 0.657). The solid line in the funnel plot represents the pooled estimate from the meta-analysis, while the dotted line the meta-regression for sqrt(sample size).

No impact of the sample size on the dose-response curve was detected (χ^2=0.84, df=2, p=0.66).

Risperidone

Forest plot

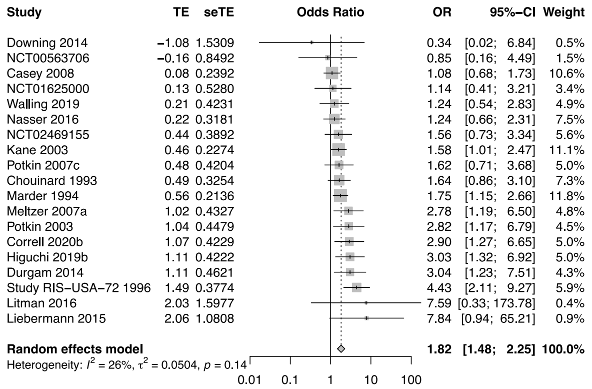

Funnel plot

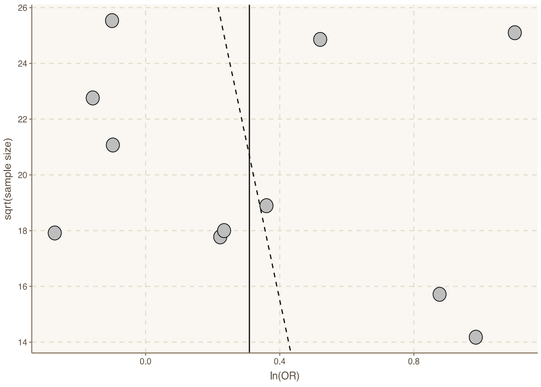

There were 19 placebo-controlled studies. No clear asymmetry was detected in the funnel plot and meta-regression (z-value=0.935, p-value = 0.350). The solid line in the funnel plot represents the pooled estimate from the meta-analysis, while the dotted line the meta-

No impact of the sample size on the dose-response curve was detected (χ^2=3.7, df=2, p=0.16).

References

1. Peters JL, Sutton AJ, Jones DR, Abrams KR, Rushton L (2006): Comparison of two methods to detect publication bias in meta-analysis. *Jama*. 295:676-680.
2. Salanti G, Peter N, Tonia T, Holloway A, White IR, Darwish L, et al. (2022): The Impact of the COVID-19 Pandemic and Associated Control Measures on the Mental Health of the General Population : A Systematic Review and Dose-Response Meta-analysis. *Ann Intern Med*.
3. Crippa A, Orisini N (2016): Multivariate Dose-Response Meta-Analysis: The dosresmeta R Package. *Journal of Statistical Software, Code Snippets*. 72:1-15.

eAppendix-9: Confidence in the evidence using the GRADE approach

Approach

We used an adapted version of the GRADE approach (1) to evaluate confidence in the evidence of the dose-response curves considering the domains of risk of bias, reporting bias, indirectness, inconsistency, and imprecision. A similar adaption was used in a previous analysis in order to evaluate the confidence in the evidence of a dose-response curve across all doses (2).

The confidence in the evidence could be rated as very low, low, moderate or high. The confidence in the evidence for each dose-response curve started from a high level and its confidence was downrated by one or two levels due to concerns in the above-mentioned domains and according to the following criteria.

Risk of bias

We evaluated the overall risk of bias of each study as low, unclear or high using the Cochrane risk of bias tool version 1 and an algorithm of a previous review to evaluate the overall risk of bias (3) (see eAppendix-5). Then, we calculated the percentage of studies with moderate or high overall risk of bias that contributed to a dose-response analysis. We downrated by one level, when the percentage was between 50-75% and by two levels when it was >75%.

Reporting bias

A priori we considered that there is a small chance that included RCTs were selected based on their results on extrapyramidal side-effects. We downrated the confidence in the evidence by one level when there were concerns of reporting bias as identified in the dose-response meta-regression using sample size as a covariate, i.e., for lurasidone (eAppendix-9).

Indirectness

There was no concern of indirectness, and thus, the confidence in the evidence was not downrated accordingly for this domain.

Inconsistency

Heterogeneity was quantified using the VPC (eAppendix-8), and confidence in the evidence was downrated by one level when the median VPC was 50-75%, and two levels when it was >75%.

Impression

We assessed imprecision across the dose-response curve. We considered the average width of the 95%CIs, and when the average width was wide (more than twice a small effect size after transforming ORs to SMDs), we downrated by one level (e.g., when $OR_{upper\text{-}boundary}/OR_{lower\text{-}boundary}>2.06$) or two levels (when $OR_{upper\text{-}boundary}/OR_{lower\text{-}boundary} >4.25$). When there was no imprecision according to this approach, but the total number of participants was <800, then imprecision was downrated by one level.

Reference

1. Guyatt GH, Oxman AD, Vist GE, Kunz R, Falck-Ytter Y, Alonso-Coello P, et al. (2008): GRADE: an emerging consensus on rating quality of evidence and strength of recommendations. *Bmj*. 336:924-926.
2. Salanti G, Peter N, Tonia T, Holloway A, White IR, Darwish L, et al. (2022): The Impact of the COVID-19 Pandemic and Associated Control Measures on the Mental Health of the General Population : A Systematic Review and Dose-Response Meta-analysis. *Ann Intern Med*.
3. Furukawa TA, Salanti G, Atkinson LZ, Leucht S, Ruhe HG, Turner EH, et al. (2016): Comparative efficacy and acceptability of first-generation and second-generation antidepressants in the acute treatment of major depression: protocol for a network meta-analysis. *BMJ open*. 6:e010919.

Confidence in the evidence for dose-response curves of individual antipsychotics

Patient subgroup	Drug	n studies	N participants	k arms	% moderate or high risk of bias	Width of the 95%CI (OR_{upper}/OR_{lower})	Median VPC (%)	Risk of bias	Reporting bias	Indirectness	Imprecision	Inconsistency	Confidence in the evidence
Negative symptoms	Amisulpride	4	591	10	100	0.99	0	Major concerns	No concerns	No concerns	Major concerns	No concerns	Very Low
	Olanzapine	1	174	3	100	1.07	n.a.	Major concerns	No concerns	No concerns	Major concerns	No concerns	Very Low
Chronically-ill patients with acute exacerbation	Amisulpride	1	255	4	0	0.97	n.a.	No concerns	No concerns	No concerns	Major concerns	No concerns	Low
	Aripiprazole	9	2259	26	44.44	0.5	52	No concerns	No concerns	No concerns	Some concerns	Some concerns	Low
	Asenapine	6	2242	17	33.33	0.44	24.2	No concerns	No concerns	No concerns	Some concerns	No concerns	Moderate
	Brexpiprazole	4	2178	17	0	0.27	0	No concerns	No concerns	No concerns	No concerns	No concerns	High
	Cariprazine	5	1918	17	100	0.57	45.6	Major concerns	No concerns	No concerns	Some concerns	No concerns	Very Low
	Haloperidol	17	2623	38	47.06	0.45	48.2	No concerns	No concerns	No concerns	Some concerns	No concerns	Moderate
	Iloperidone	2	952	6	50	0.98	0	Some concerns	No concerns	No concerns	Major concerns	No concerns	Very Low
	Lumateperone	3	1225	9	33.33	1.22	7.8	No concerns	No concerns	No concerns	Major concerns	No concerns	Low
	Lurasidone	11	3649	32	9.09	0.44	13.6	No concerns	Some concerns	No concerns	Some concerns	No concerns	Low

	Olanzapine	16	3813	40	37.5	0.58	20.4	No concerns	No concerns	No concerns	Some concerns	No concerns	Moderate
	Paliperidone	11	4215	36	18.18	0.31	35	No concerns	No concerns	No concerns	No concerns	No concerns	High
	Quetiapine	9	3058	32	33.33	0.59	30.7	No concerns	No concerns	No concerns	Some concerns	No concerns	Moderate
	Risperidone	23	6151	60	43.48	0.26	22.4	No concerns	No concerns	No concerns	No concerns	No concerns	High
	Sertindole	4	1332	15	25	0.41	0	No concerns	No concerns	No concerns	Some concerns	No concerns	Moderate
	Ziprasidone	8	1785	25	62.5	0.51	28.9	Some concerns	No concerns	No concerns	Some concerns	No concerns	Low
	Zotepine	2	119	4	50	3.09	40.9	Some concerns	No concerns	No concerns	Major concerns	No concerns	Very Low
First-episode patients	Risperidone	2	72	4	50	3.09	40.9	Some concerns	No concerns	No concerns	Major concerns	No concerns	Very Low

eAppendix-10: Relationship between the D_2R occupancy and the risk of EPS

Individual antipsychotics

We used the dose-response curves of the individual antipsychotics (as presented in Figure-2), and converted the daily dose to the D_2R occupancy using the formulas presented in a previous meta-analysis (1). The confidence in the evidence was further downrated by one level due to some concerns in indirectness given that the D_2R occupancy was estimated from daily doses, i.e., plasma concentrations correlate better than daily dose (2).

The findings were generally in accordance with the D_2R therapeutic window of dopamine 2 receptor antagonists, showing that the risk of EPS is increasing substantially at D_2R occupancies >80%. This was clear for haloperidol, olanzapine and risperidone. Amisulpride and ziprasidone showed a higher risk of EPS at smaller occupancies, but their results were based on very low quality of evidence. Quetiapine did not occupy importantly D_2R (<50%) and had a low risk of EPS. Aripiprazole had also a lower risk of EPS even at D_2R occupancies higher than 80%, given that it acts as a dopamine partial agonist.

Combined D_2R antagonists

We aimed to further explore the relationship between D_2R occupancy and the risk of EPS, given that similar relationships were observed for the individual D_2R antagonists. Thus, we converted daily doses to D_2R occupancies, and we conducted a dose-response meta-analysis by combining six antipsychotics, i.e., amisulpride, haloperidol, olanzapine, paliperidone, quetiapine, risperidone, ziprasidone. We did not include aripiprazole, given that it is a D_2R partial agonist with a clearly different relationship between D_2R occupancy and ORs for EPS. In this analysis, we set knot points at 25th, 50th, 95th of D_2R occupancies >50%, given that we expect changes at this part of the curve (3).

The findings showed that the risk of EPS increased substantially at D_2R occupancies about >75-85% (Figure-4). There was low confidence in the evidence according to the GRADE approach.

Confidence in the evidence using the GRADE approach for relationship between D_2R occupancy and risk of EPS (D_2R antagonists combined, thus not including aripiprazole)

n studies	N participants	k arms	Risk of bias	Reporting bias	Indirectness	Imprecision	Inconsistency	Confidence in the evidence
68	17396	194	No concerns	No concerns	Major concerns	No concerns	No concerns	Low

There were no concerns in terms of risk of bias (42.7% of the studies had an overall moderate or high risk of bias), reporting bias (Wald test of the coefficients for sample size χ^2=0.52, df=2, p-value=0.77), imprecision (narrow confidence intervals and the average width was OR_{upper}/OR_{lower}=1.52) and inconsistency (median VPC=48.1%, see below the plot). However, there were major concerns in indirectness given that the

D_2R occupancy was estimated from daily doses (plasma concentrations correlate better than daily dose (2)), and because there were available data only for six antipsychotics.

VPC for the D_2R occupancy analysis for combined antipsychotics

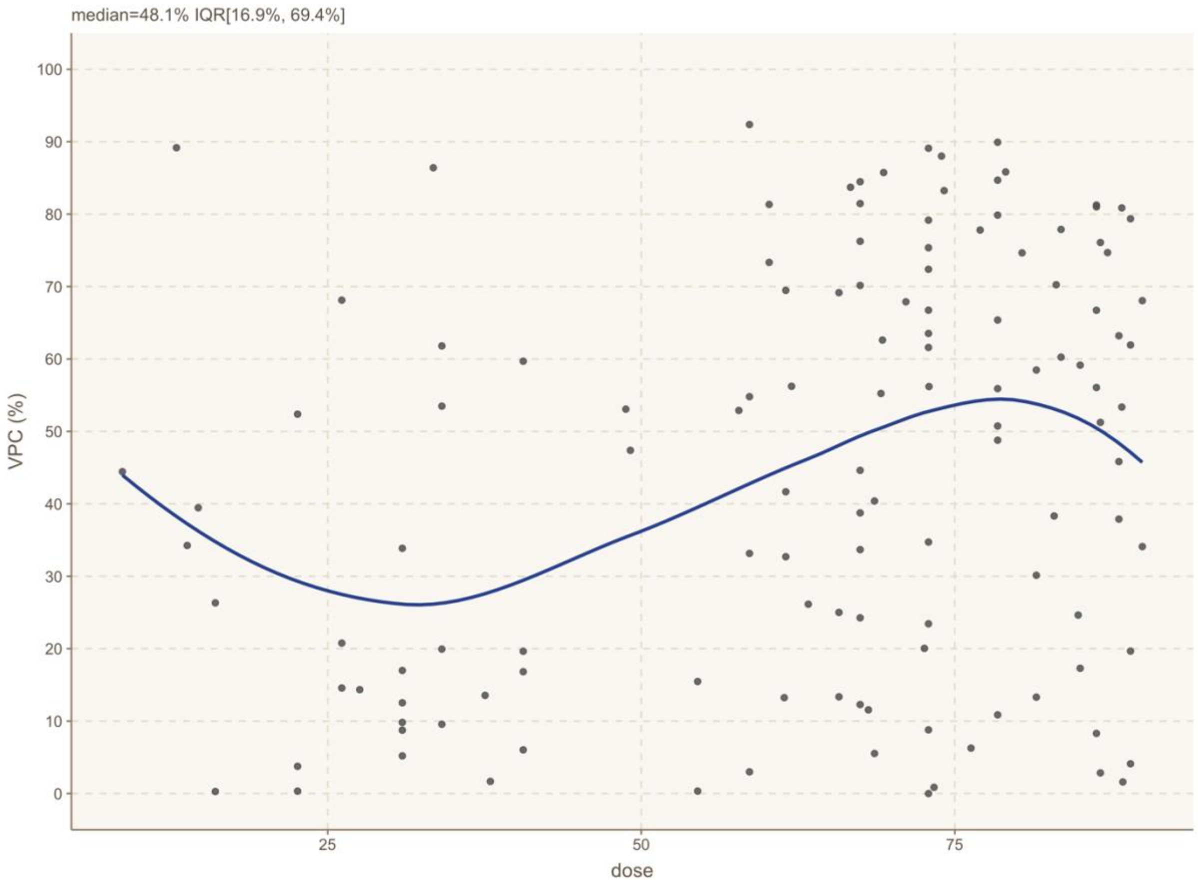

References

1. Lako IM, van den Heuvel ER, Knegtering H, Bruggeman R, Taxis K (2013): Estimating dopamine D_2 receptor occupancy for doses of 8 antipsychotics: a meta-analysis. *J Clin Psychopharmacol*. 33:675-681.

2. Hiemke C, Bergemann N, Clement HW, Conca A, Deckert J, Domschke K, et al. (2018): Consensus Guidelines for Therapeutic Drug Monitoring in Neuropsychopharmacology: Update 2017. *Pharmacopsychiatry*. 51:e1.

3. de Greef R, Maloney A, Olsson-Gisleskog P, Schoemaker J, Panagides J (2011): Dopamine D2 occupancy as a biomarker for antipsychotics: quantifying the relationship with efficacy and extrapyramidal symptoms. *The AAPS journal*. 13:121-130.

eAppendix-11: Risk of EPS at recommended and near-maximal effective doses of antipsychotics

To interpret the risk of extrapyramidal side-effects (EPS) associated with a dose of an antipsychotic, it is important to consider the dose-response relationships for efficacy. To facilitate this, we plotted dose-response curves, similar to Figure-2, highlighting recommended dose ranges, and provided a table presenting in more detail their corresponding risk of EPS.
We presented findings for chronically-ill patients with an acute exacerbation, and not for the other patient subgroups (predominant negative symptoms, first-episode) due to their limited available data.

We referred to Table 1 in Leucht et al 2020 (1) regarding the recommended dose ranges and the 95% effective dose (ED95) for the individual antipsychotics: The recommended dose ranges (minimum target dose and maximum approved dose) were extracted from summary produce characteristics (SPC), while the ED95 was calculated with a two-stage dose-response meta-analysis. The ED95 is defined as the mean dose that achieves 95% of the maximum efficacy (near-maximum dose) of the antipsychotic in terms of reducing overall symptoms.

For zotepine, an ED95 value was not available, as well as we extracted its recommended dose range from the international consensus of Gardner et al 2010 (2). Additionally, we presented the recommended doses for quetiapine immediate release (150-750mg/d for immediate release, 400-800mg/d for extended release).

It is important to acknowledge that the recommended dose ranges for antipsychotic medications can vary across different clinical practice guidelines. Therefore, the dose ranges used in this study were only intended to provide a framework for interpreting the risk of extrapyramidal side-effects (EPS).

Dose-response curves for EPS and recommended dose ranges of antipsychotics

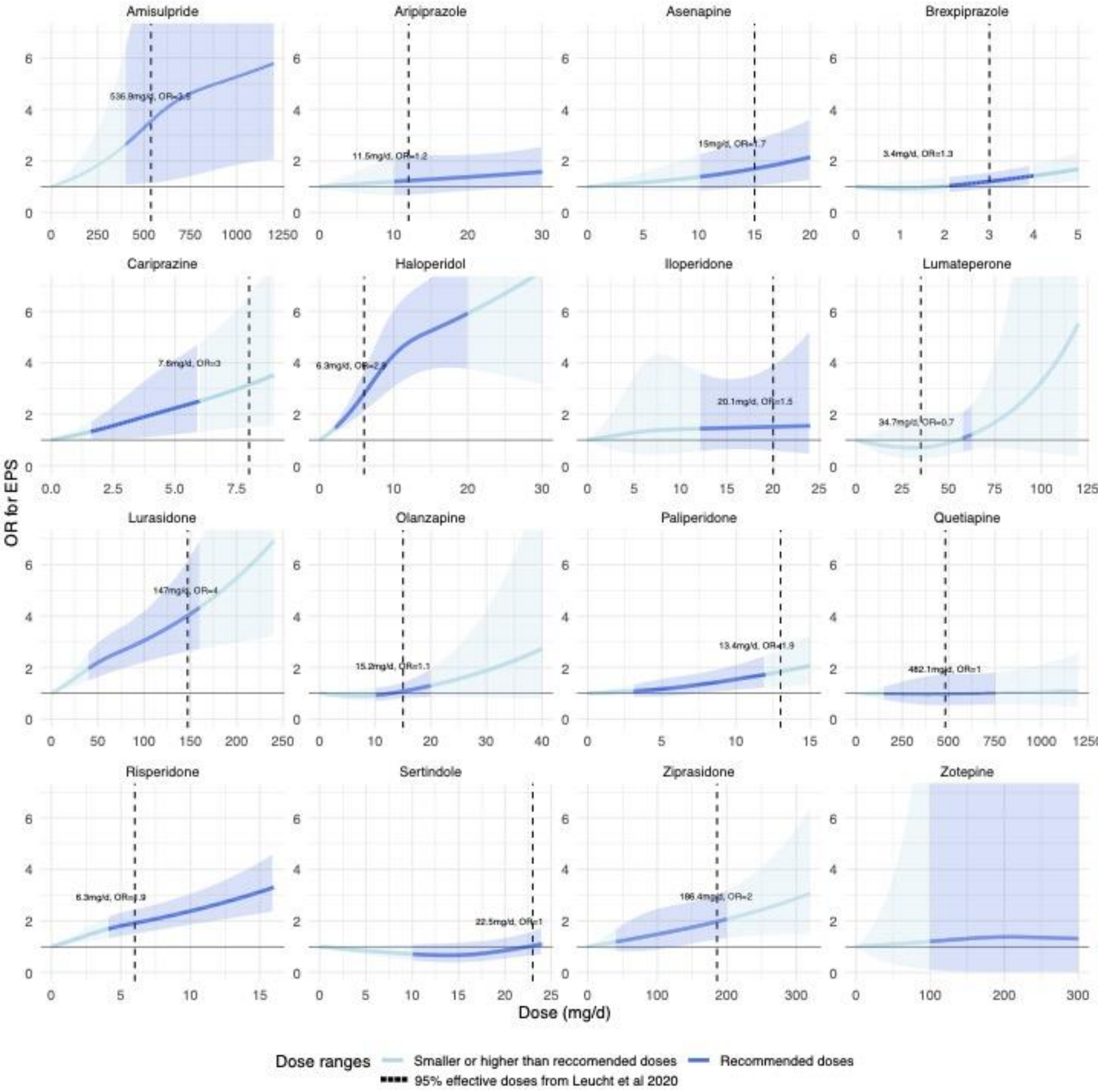

The dose-response curves show the risk of EPS for doses (mg/d) of individual antipsychotics. The risk is quantified with ORs using placebo (0mg/d) as reference. The recommended doses are highlighted with blue, doses outside this range with light blue and the near-maximal or 95% effective doses (ED95) with dashed line. The ED95 of an antipsychotic is the mean dose that achieves the 95% of the maximum efficacy of the antipsychotic in terms of reduction of the overall symptoms.

Table of the risk for EPS at recommended and near-maximal doses of antipsychotics

	Near-maximal or 95% effective dose (ED95) from Leucht et al 2020 (1)			Recommended doses						Maximum dose above recommended with available data		
				Minimum target dose			Maximum recommended dose					
**Antipsychotic**	**Dose (mg/d)**	**OR (95%CI)**		**Dose (mg/d)**	**OR (95%CI)**		**Dose (mg/d)**	**OR (95%CI)**		**Dose (mg/d)**	**OR (95%CI)**	
Amisulpride	536.9	3.5	(1.1, 10.9)	400	2.6	(1.1, 6.4)	1200	5.8	(2.1, 16.3)	n.a		
Aripiprazole	11.5	1.2	(0.7, 2.2)	10	1.2	(0.7, 2.1)	30	1.6	(1, 2.6)	n.a		
Asenapine	15	1.7	(1, 2.8)	10	1.4	(0.8, 2.2)	20	2.1	(1.3, 3.6)	n.a		
Brexpiprazole	3.4	1.3	(1, 1.7)	2	1.0	(0.8, 1.4)	4	1.4	(1.1, 1.9)	5	1.7	(1.2, 2.3)
Cariprazine	7.6	3.0	(1.5, 6.2)	1.5	1.3	(1, 1.7)	6	2.5	(1.3, 4.8)	9	3.5	(1.6, 8)
Haloperidol	6.3	2.9	(2.2, 3.9)	2	1.4	(1.3, 1.6)	20	5.9	(3.8, 9.3)	30	7.6	(3.2, 18.1)
Iloperidone	20.1	1.5	(0.6, 3.9)	12	1.4	(0.6, 3.6)	24	1.6	(0.5, 5.3)	n.a		
Lumateperone	34.7	0.7	(0.3, 1.9)	60	1.1	(0.6, 2.2)	60	1.1	(0.6, 2.2)	120	5.5	(0.4, 80.5)
Lurasidone	147	4.0	(2.6, 6.2)	40	2.0	(1.5, 2.6)	160	4.3	(2.7, 6.9)	240	6.9	(3.2, 14.8)
Olanzapine	15.2	1.1	(0.8, 1.4)	10	0.9	(0.7, 1.2)	20	1.3	(0.9, 2)	40	2.7	(0.8, 9.5)
Paliperidone	13.4	1.9	(1.3, 2.8)	3	1.1	(0.9, 1.3)	12	1.7	(1.2, 2.5)	15	2.1	(1.3, 3.3)
Quetiapine*	482.1	1.0	(0.5, 1.7)	150	1.0	(0.8, 1.3)	750	1.0	(0.6, 1.8)	1200	1.1	(0.5, 2.6)
Risperidone	6.3	1.9	(1.5, 2.5)	4	1.7	(1.3, 2.1)	16	3.3	(2.4, 4.6)	n.a		
Sertindole	22.5	1.0	(0.6, 1.5)	10	0.7	(0.5, 1.1)	24	1.1	(0.7, 1.7)	n.a		
Ziprasidone	186.4	2.0	(1.3, 3)	40	1.2	(0.8, 1.7)	200	2.1	(1.3, 3.2)	320	3.1	(1.5, 6.4)
Zotepine	n.a.			100	1.2	(0.1, 13.6)	300	1.3	(0, 70.7)	n.a		

n.a.: not available data, *The recommended doses for quetiapine immediate release are presented.

References

1. Leucht S, Crippa A, Siafis S, Patel MX, Orsini N, Davis JM (2020): Dose-Response Meta-Analysis of Antipsychotic Drugs for Acute Schizophrenia. *Am J Psychiatry*. 177:342-353.
2. Gardner DM, Murphy AL, O'Donnell H, Centorrino F, Baldessarini RJ (2010): International consensus study of antipsychotic dosing. *Am J Psychiatry*. 167:686-693.

eAppendix-12: Abbreviations

Abbreviation	Description
5-HT_{1A}	5-hydroxytryptamine 1A receptor
5-HT_{2A}	5-hydroxytryptamine 2A receptor
5-HT_{2c}	5-hydroxytryptamine 2c receptor
CENTRAL	Cochrane Central Register of Controlled Trials
CSzG	Cochrane Schizophrenia Group
D_2R	Dopamine 2 receptor
DSM	Diagnostic and Statistical Manual of Mental Disorders
ED95	95% effective dose or near-maximal doses, i.e., dose that achieves the 95% of the maximum efficacy
EPS	Extrapyramidal side-effects
GRADE	Grading of Recommendations Assessment, Development and Evaluation
IQR	Interquartile range
IR	Immediate release
LAI	Long-acting injectable
LOCF	Last-observation carried forward
M_1	M1 muscarinic acetylcholine receptor
mg/d	Milligram per day
MMRM	Mixed-models of repeated measurement
n.a.	Not available
OR	Odds ratio
PRISMA	Preferred Reporting Items for Systematic Reviews and Meta-Analyses
RCT	Randomized controlled trial
SAS	Simpson Angus Scale
SD	Standard deviation
SGA	Second generation antipsychotic
SMD	Standardized mean difference
sqrt	Squared
VPC	Variance partition coefficients
XR	Extended release
